# The Arbuscular Mycorrhizal Fungus *Rhizophagus irregularis* MUCL 41833 Modulates Metabolites Production of *Anchusa officinalis* L. Under Semi-Hydroponic Cultivation

**DOI:** 10.3389/fpls.2021.724352

**Published:** 2021-09-01

**Authors:** Annalisa Cartabia, Evangelia Tsiokanos, Nikolaos Tsafantakis, Ismahen Lalaymia, Aikaterini Termentzi, Maria Miguel, Nikolas Fokialakis, Stéphane Declerck

**Affiliations:** ^1^Applied Microbiology, Mycology, Earth and Life Institute, Université catholique de Louvain, Louvain-la-Neuve, Belgium; ^2^Department of Pharmacognosy and Natural Product Chemistry, Faculty of Pharmacy, National and Kapodistrian University of Athens, Athens, Greece; ^3^Laboratory of Pesticides' Toxicology, Department of Pesticides Control and Phytopharmacy, Benaki Phytopathological Institute, Athens, Greece; ^4^Instituto de Tecnologia Química e Biológica António Xavier, Universidade Nova de Lisboa (ITQB NOVA), Oeiras, Portugal

**Keywords:** arbuscular mycorrhizal fungi, *Rhizophagus irregularis*, *Anchusa officinalis*, semi-hydroponic cultivation system, metabolomics, primary and secondary metabolites

## Abstract

*Anchusa officinalis* is recognized for its therapeutic properties, which are attributed to the production of different metabolites. This plant interacts with various microorganisms, including the root symbiotic arbuscular mycorrhizal fungi (AMF). Whether these fungi play a role in the metabolism of *A. officinalis* is unknown. In the present study, two independent experiments, associating *A. officinalis* with the AMF *Rhizophagus irregularis* MUCL 41833, were conducted in a semi-hydroponic (S-H) cultivation system. The experiments were intended to investigate the primary and secondary metabolites (PMs and SMs, respectively) content of shoots, roots, and exudates of mycorrhized (M) and non-mycorrhized (NM) plants grown 9 (Exp. 1) or 30 (Exp. 2) days in the S-H cultivation system. Differences in the PMs and SMs were evaluated by an untargeted ultrahigh-performance liquid chromatography high-resolution mass spectrometry metabolomics approach combined with multivariate data analysis. Differences in metabolite production were shown in Exp. 1. Volcano-plots analysis revealed a strong upregulation of 10 PMs and 23 SMs. Conversely, in Exp. 2, no significant differences in PMs and SMs were found in shoots or roots between M and NM plants whereas the coumarin scoparone and the furanocoumarin byakangelicin, accumulated in the exudates of the M plants. In Exp. 1, we noticed an enhanced production of PMs, including organic acids and amino acids, with the potential to act as precursors of other amino acids and as building blocks for the production of macromolecules. Similarly, SMs production was significantly affected in Exp 1. In particular, the phenolic compounds derived from the phenylpropanoid pathway. Fifteen di-, tri-, and tetra-meric C_6_-C_3_ derivatives of caffeic acid were induced mainly in the roots of M plants, while four oleanane-types saponins were accumulated in the shoots of M plants. Two new salvianolic acid B derivatives and one new rosmarinic acid derivative, all presenting a common substitution pattern (methylation at C-9”' and C-9' and hydroxylation at C-8), were detected in the roots of M plants. The accumulation of diverse compounds observed in colonized plants suggested that AMF have the potential to affect specific plant biosynthetic pathways.

## Introduction

The plant kingdom is an incredible source of structurally and functionally diverse metabolites (e.g., phenolic compounds, amino acids, peptides, terpenes, and alkaloids) useful for human life (Wang et al., 2019). Many of these (called primary or secondary metabolites, PMs and SMs, respectively) are of commercial interest, and currently exploited in fields such as cosmetics, pharmaceuticals, nutraceuticals, agrochemicals, food and fine chemicals (Charles Dorni et al., 2017; Neelam and Sharma, 2020). Interestingly, plants have evolved with highly complex assemblages of microorganisms (called the microbiota), having roles in plant development and health (Rivero et al., 2015). Many of these microorganisms produce metabolites of interest but, in numerous cases, also influence the metabolism of plants and thus their qualitative and quantitative production of metabolites (Kaur and Suseela, 2020).

Among the microorganisms that interact intimately with plants are the arbuscular mycorrhizal fungi (AMF). These soil fungi, belonging to the Glomeromycota Phylum, are obligate symbionts that live in association with nearly 72% of plant species (Brundrett and Tedersoo, 2018). They provide their hosts with nutrients (especially N and P) in exchange for carbon and lipids (Smith et al., 2011; Chen et al., 2018) and increase their resistance to a/biotic stresses (Gianinazzi et al., 2010).

In the recent decade, a number of studies have reported the impact of AMF on the production of plant PMs, and on various biosynthetic pathways involved in the production of SMs in the leaves, roots, or fruits/tubers of different crops used as food or for medicinal purposes (Gianinazzi et al., 2010; Zeng et al., 2013; Pedone-Bonfim et al., 2015; Pandey et al., 2018). Most studies involving metabolic analysis of AMF-colonized plants were focused on few chemical groups, and it is only in the last decade that the first untargeted metabolomics analysis, allowing a simultaneous detection of a wide variety of compounds, was conducted (Rivero et al., 2015; Hill et al., 2018). Interestingly, most of these studies reported a non-negligible impact of AMF on the production of PMs and SMs by plants. For instance, Schliemann et al. (2008) investigated the metabolic changes in the roots of the model plant *Medicago truncatula* colonized with *Rhizophagus irregularis*. Their study, conducted over a nearly 2-month period, showed a strong effect of AMF on polar and nonpolar PMs and SMs. More precisely, PMs such as amino acids, fatty acids or alcohols and alkanes, and SMs, such as isoflavonoids, phenylpropanoid derivatives, and apocarotenoids, were accumulated in higher proportion in the roots of AMF-colonized plants than in those of the non-colonized controls. More recently, Saia et al. (2015) have shown a decrease in amino acids and saturated fatty acids content of *Triticum durum* roots inoculated with a pool of different AMF species, while Rivero et al. (2015) presented through a detailed metabolomics analysis clear metabolites differences in tomato roots associated with two different AMF species (*Funneliformis mosseae* and *R. irregularis*). Their untargeted metabolomics analysis showed a notable increase of several signals referring to sugars, carboxylic acids, amino acids, and compounds from the phenylpropanoid pathway in AMF-colonized versus non-colonized plants. Regarding the aboveground metabolome, the analysis of leaves of several plants, both dicots and monocots, colonized by *R. irregularis*, has shown an up/downregulation of different PMs (e.g., amino acids, organic acids) and SMs (e.g., catalpol, verbascoside); hence, many species-specific responses were reported (Schweiger et al., 2014). The majority of the studies considered the foliar or the aboveground metabolic variations, without taking into consideration both parts (shoot and roots) concomitantly. For this reason, Hill et al. (2018) investigated the metabolites profile in both foliar and root tissues of *Senecio jacobae*. Their untargeted metabolic approach revealed a significant upregulation of SMs belonging mainly to blumenol derivates and anti-herbivore defense compounds as pyrrolizidine alkaloids. Nevertheless, the changes were found mostly in the root system, while a slight or nonsignificant metabolic modification was observed in the shoot parts of the plants.

So far, the metabolomics studies on plants associated with AMF were, to the best of our knowledge, conducted in pot cultures or field trials under semi-controlled conditions. However, for medicinal plants, metabolites production is more often conducted under highly controlled and up-scalable conditions, such as bioreactors or hydroponic cultivation systems (Gontier et al., 2002; Sgherri et al., 2010; Malik et al., 2016), both being never applied to AMF-medicinal plant associates. Therefore, moving to hydroponics could represent a step forward to study the metabolomics reprogramming of plants (medicinal or others) associated with AMF under highly controlled conditions.

Recently, a semi-hydroponic (S-H) cultivation system with perlite as inert substrate has been developed by Garcés-Ruiz et al. (2017) to study the dynamics of inorganic phosphorus (Pi) uptake from a circulating nutrient solution by maize plants colonized with the AMF *R. irregularis* MUCL 41833. After 25 days of experiment, a considerable increase in plant growth, Pi uptake, and strong root colonization (e.g., 90%) was observed, suggesting that the S-H cultivation system is adequate for growing AMF-colonized plants.

*Anchusa officinalis* L. belongs to the Boraginaceae family. This plant is mostly encountered in the southern part of the Balkan Peninsula (Selvi and Bigazzi, 1998) in sunny warm places, such as fields, meadows, and river sediments. A phytochemical screening, performed on wild plants by Jakovljevic et al. (2016) and Boskovic et al. (2018), demonstrated the presence of an abundant content of phenolic compounds, especially caffeic acid esters such as rosmarinic acid (RA). This compound presents several health-related properties (Dresler et al., 2017), conferring antioxidant, antibacterial, and anti-inflammatory activities to *A. officinalis* extracts. The association between *A. officinalis* and AMF has been reported by Zubek et al. (2011), but no study has ever reported the effects of these beneficial soil-borne fungi on the metabolites profile of this plant.

In the present study, two independent experiments, associating *A. officinalis* with the AMF *R. irregularis* MUCL 41833, were conducted with the S-H cultivation system described above. The experiments were intended to investigate the PMs and SMs content of shoots, roots, and exudates of mycorrhized (M) or non-mycorrhized (NM) plants grown 9 (Exp. 1) or 30 (Exp. 2) days in the S-H cultivation system. In both experiments, ultrahigh-performance liquid chromatography high-resolution mass spectrometry (UHPLC-HRMS) was used. An untargeted metabolomics approach was further conducted to shed light on the overall effects of AMF on *A. officinalis* plants grown under highly controlled S-H culture conditions.

## Materials and Methods

### Chemicals

HPLC grade methanol (MeOH) was obtained from Fisher Chemical (Fisher Scientific, Loughborough, UK), acetonitrile LC-MS grade (LiChrosolv^®^ hypergrade) (ACN), and formic acid LC-MS grade (LiChropur^®^) (FA) from Merck (Merck KGaA, Darmstadt, Germany) and ethyl acetate (ExpertQ^®^, 99.8%) (EtOAc) from Scharlau Basic (a.r. grade, Scharlab S.L., Barcelona, Spain). Ultrapure water was received from LaboStar apparatus (Evoqua LaboStar^®^ 4, Evoqua Water Technologies, Pittsburgh, USA). Discovery^®^ DSC-C18 Supelco SPE cartridges of 500 mg bedweight and 6-ml volume were purchased from Sigma-Aldrich (Taufkirchen, Germany).

### Biological Material

Seeds of *Anchusa officinalis* L. were provided by Rühlemann's herbs and aromatic plants (Germany). They were surface-disinfected by immersion in sodium hypochlorite (8% active chloride) for 5 min and rinsed three times with sterilized (121°C for 15 min) deionized water. The seeds were then germinated in plastic seed trays (37.5 × 23 × 6 cm) filled with a mix (w/w 1:2) of sterilized (121°C for 15 min) perlite (Perligran Medium, KNOUF-GMB, Germany) and turf (DCM, Belgium). The trays were placed in the greenhouse set at 25°C/18°C (day/night), relative humidity (RH) of 38%, a photoperiod of 16-h day^−1^ and photosynthetic photon flux (PPF) of 120 μmol m^−2^ s^−1^.

The AMF *Rhizophagus irregularis* [Błaszk, Wubet, Renker, and Buscot) C. Walker and A. Schüßler as (“irregulare”)] MUCL 41833 was supplied by the Glomeromycota *in vitro* collection (GINCO)^1^. The fungus was proliferated on plants of *Zea mays* L. cv. ES Ballade (Euralis, France) in a 10-L plastic box containing sterilized (121°C for 15 min) lava (DCM, Belgium). The plants were grown under the same greenhouse conditions as above.

### Colonization of *A. officinalis*

Two-week-old *A. officinalis* plants were transferred in 10-L pots containing a sterilized (121°C for 15 min) mix of lava and perlite (w/w 2:1). For the mycorrhizal (M) treatment, the substrate was half mixed with the AMF-inoculum substrate above (final-ratio lava: perlite w/w 5:1). For the non-mycorrhizal (NM) treatment (i.e., the control), the substrate was half mixed with the AMF-inoculum substrate above but sterilized (121°C for 15 min) in the same final ratio (5:1). The plants were grown under the same greenhouse conditions as above.

### Metabolites Profile of *A. officinalis* Associated With *R. irregularis* in a 9-Day Experiment Conducted in a S-H Cultivation System (Experiment 1)

#### Experimental Setup

In the first experiment, the objective was to evaluate the metabolites profile of M and NM *A. officinalis* plants grown for 9 days in the S-H cultivation system. The metabolites released in the circulating nutrient solutions were also assessed.

**Figure 1:**
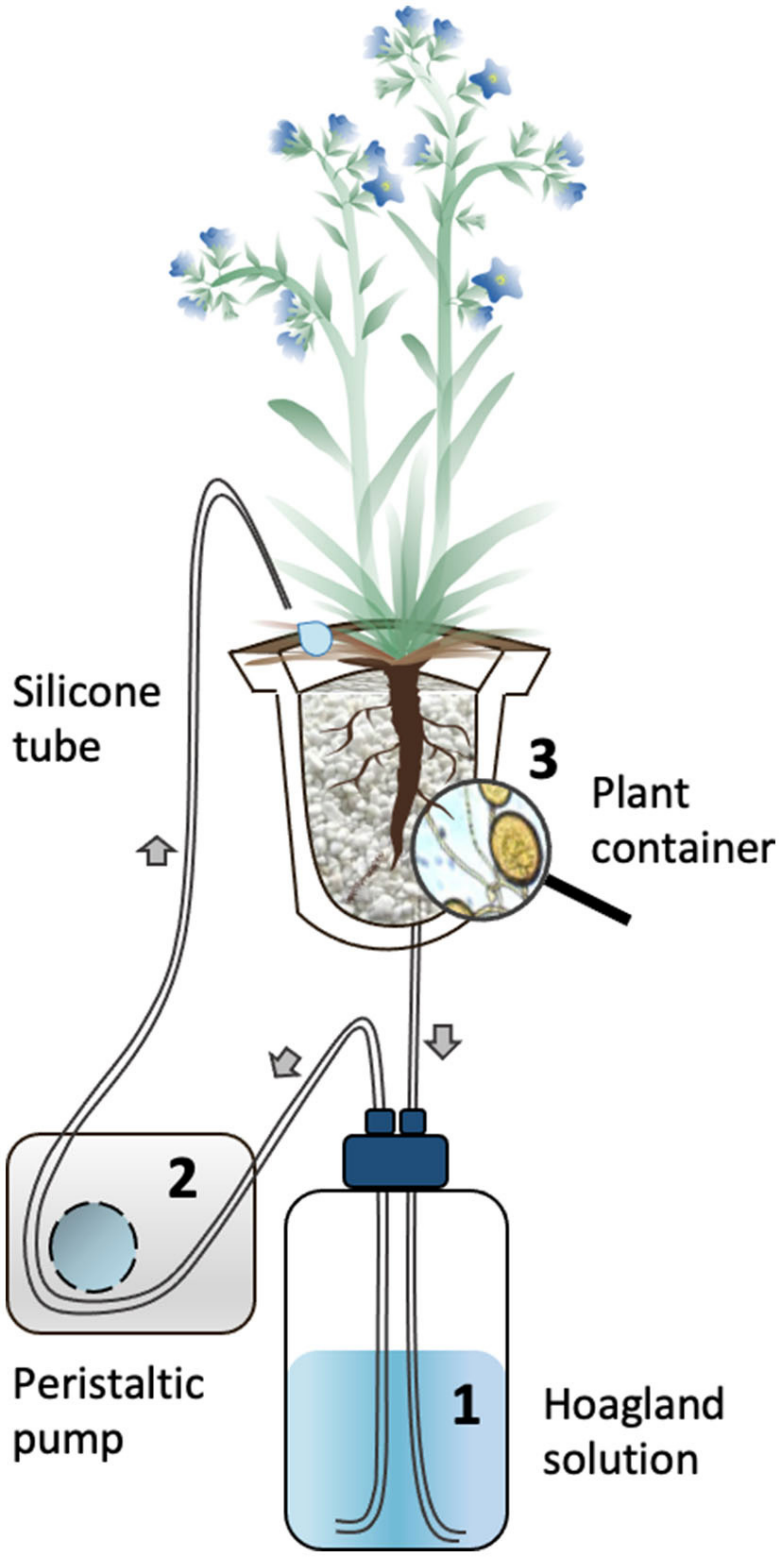
Schematic representation of the circulatory S-H cultivation system. The Hoagland solution circulated through the containers supporting mycorrhized (M) and non-mycorrhized (NM) *A. officinalis* plants. The nutrient solution in the glass bottle (1) is pumped using a peristaltic pump (2) to the upper part of the plant container (3) *via* silicon tubes. The solution percolate through the plant container back into the glass bottle. The black arrows indicate the flow direction of the nutrient solution in the tubing.

Two-month-old plants (7 M and 6 NM) were gently removed from the 10-L pots above and their roots cleaned with deionized water to eliminate lava and perlite debris. They were subsequently transferred to the S-H cultivation system (Figure 1) (see for details Garcés-Ruiz et al., 2017) as follows: The plants were placed in 500-ml wash plastic bottles (VWR, USA) cut at the base and placed bottom-up. A 100-μm size pore nylon mesh (Prosep B.V.B.A., Belgium) was glued on the top of the bottles (called containers thereafter) to prevent loss of substrate and roots growing out of the containers. Each container was filled with 32 g of sieved (1-mm diameter), rinsed (with deionized water), and dried (48 h at 50°C) perlite (KNOUF GMBH, Germany). The perlite was covered with a superficial layer of black lava rock (1–3 mm) and containers wrapped in aluminum foil to avoid algae development. The containers were transferred randomly in holes made in flex foam supports and were maintained in the greenhouse set at the same conditions as described above.

A 90% P-impoverished modified Hoagland solution [see Garcés-Ruiz et al. (2017)] was used at two different concentrations: diluted by 200× (referred as Hoagland^dil200X^) during the acclimatization phase (7 days) and diluted by 100× (referred as Hoagland^dil100X^) during the circulation phase (42 h).

The M and NM plants were acclimatized for 7 days, receiving 200-ml Hoagland^dil200X^ solution every 48 h. The containers were closed with screw caps at the bottom, which were opened in order to discard the old solution before replacement with fresh solution. After this acclimatization phase, the circulatory system was started. Each container was connected to a 1-L glass bottle (SCHOTT DURAN, Germany), covered with aluminum foil containing Hoagland^dil100X^ solution. A 3-mm diameter silicon tube (VWR, United States) connected a dropper cap fixed on the bottom of the plant container with the glass bottle, and another silicon tube of the same diameter connected the glass bottle to the upper part of the plant container *via* a multichannel peristaltic pump (Gilson's Minipuls Evolution, France). Once the circulation started, the nutrient solution was pumped from the glass bottle into the plant container and the liquid flowed back by gravity into the bottle.

Before starting with the circulation, initial flushing was performed, as described by Garcés-Ruiz et al. (2017), in order to establish an equal nutrient concentration in all the containers. Each plant container received 200-ml of Hoagland^dil100x^ solution, which was circulated at a velocity of 44-ml min^−1^ through the containers and then discarded. After this initial flushing, a regular circulation was initiated and maintained at 7.5-ml min^−1^ for 42 h (T1).

### The Metabolites Profile of *A. officinalis* Associated With *R. irregularis* in a 30-Day Experiment Conducted in an S-H Cultivation System (Experiment 2)

#### Experimental Setup

In the second experiment, the objective was to evaluate the metabolites profile of M and NM *A. officinalis* plants grown for 30 days in the S-H cultivation system. The metabolites released in the circulating nutrient solutions were also assessed. In addition, the concentrations of Pi and ${\text{NO}}_{3}^{-}$ in the nutrient solutions were analyzed at regular intervals to ascertain the uptake by the plant-AMF associates and thus vitality of the system and to verify that the concentration of minerals remains sufficient for adequate plant growth.

The same procedure as in Exp. 1 was applied. Briefly, after cleaning the roots system, two-month-old plants (7NM and 7 M) were transferred in the S-H cultivation system, and the containers were randomly placed in the holes made in the flex foam supports. Then, after the acclimatization period (7 days), four successive circulations were performed for different durations: 42 h at day 9 (T1) and 8 h at day 15 (T2), day 22 (T3), and day 30 (T4).

#### Monitoring Minerals Depletion in Hoagland^dil100X^ Low-P Solution

In this experiment exclusively, Pi and ${\text{NO}}_{3}^{-}$ concentrations were monitored in the Hoagland^dil100X^ solution of the M and NM plants grown in the S-H cultivation system. Twenty ml of Hoagland^dil100X^ solution was sampled from each bottle after each circulation (T1, T2, T3, and T4). At each sampling time, the solution was collected in 50-ml Falcon Tubes (Sarstedt, Germany) and then stored at 4°C in the dark before proceeding with the analysis.

Concentration of Pi was evaluated *via* inductively coupled plasma atomic emission spectroscopy (ICP-AES) as described by Garcés-Ruiz et al. (2017). For ${\text{NO}}_{3}^{-}$, samples were first diluted 50 times with Milli-Q water and then analyzed *via* ionic chromatography system (IC, DIONEX, DX 120). The ${\text{NO}}_{3}^{-}$ quantification was analyzed under ultraviolet-visible (UV/VIS) spectrophotometry detection between 200 and 220 nm. The limit of detection was <100 ppb.

### Plant Harvest and AMF Root Colonization

The *A. officinalis* plants were harvested at days 9 and 30, in Exp. 1 and 2, respectively. The nutrient solutions were also kept in the bottles at 4°C in the dark before proceeding with the phytochemical content analysis.

In both experiments, total fresh weight (TFW, e.g., the sum of shoots and roots), as well as root colonization of M and NM plants, was assessed at the start and the end of the experiments. Root colonization was evaluated by McGonigle et al. (1990) on one-third of the root system. The roots were first cut into small pieces (circa, 1 cm) and placed into 50-ml Falcon tubes. Twenty-five ml of KOH 10% was added to the roots before incubation at 70°C in a hoven for 45 min. The KOH solution was then removed and replaced with HCl 1% for 1 min at room temperature. The roots were subsequently stained with ink 2% (Parker Blue Ink, United States) in HCl 1% (Walker, 2005) by placing the tubes at 70°C in the hoven for 30 min. The roots were finally rinsed with deionized water and kept in lactoglycerol (lactic acid/glycerol/distilled water, 1:1:1, v/v) before observation. For colonization assessment, the root fragments were placed on microscope slides and covered with a 40 × 22mm coverslip before observation under a bright field light microscope (Olympus BH2-RFCA, Japan) at ×10 magnification. Around 200 intersections were observed per plant. The percentage of total colonization (TC%) of roots (e.g., hyphae, arbuscules, and vesicles), and percentage of arbuscules (AC%), was further calculated.

### Analysis of PMs and SMs of *A. officinalis* Plants and Nutrient Solutions

#### Sample Preparation

The remaining two-third root systems, as well as the shoot parts of six randomly chosen M and NM plants of both experiments, were separated and subjected to freeze-drying during 24 h. The freeze-dried samples were then reduced into powder, using liquid nitrogen. Twenty mg of each plant material was subsequently subjected to a 30-min exhaustive ultrasound-assisted extraction (three cycles), using a mixture of ethyl acetate and methanol (35:65 v/v) at room temperature. The samples were finally centrifuged at 3,500 rpm for 3 min, and the supernatants of each cycle were combined.

In parallel, four randomly chosen nutrient solutions of M and NM plants from both experiments, sampled after 9 and 30 days, were prepared for analysis as follows: 50 ml of each nutrient solution was subjected to a solid phase extraction process, using a C18 cartridge (500 mg/6 ml) previously preconditioned with 6 ml of MeOH, followed by an equilibration step with 6 ml of Milli-Q water. The SPE cartridges were loaded with the nutrient solutions, washed with Milli-Q water, and dried under a slight vacuum. Finally, the elution was performed, using 12 ml of methanol, and the resulting solutions were dried under a nitrogen stream.

In total, six independent M and NM plants grown in different S-H systems and their corresponding nutrient solution eluates were considered. Each plant was analyzed in triplicate. Prior to analysis, the nutrient solution eluates and plant extracts were filtered through a 45-μm pore size hydrophilic polyvinylidene fluoride membrane. Each sample was adjusted to the final concentration of 300 μg ml^−1^, using 50% H_2_O: MeOH of LC-MS grade.

#### UHPLC-HRMS Analysis

High-resolution metabolomic profiling of M and NM samples was performed on an UHPLC-HRMS/MS Orbitrap Q-Exactive platform (Thermo Scientific San Jose, CA, USA). A full scan with a mass range of 100–1,200 Da on a centroid mode was applied, while HRMS data (70.000 resolution) were collected in both negative and positive ionization modes under the following conditions: capillary temperature, 320°C; spray voltage, 2.7 kV; S-lense Rf level, 50 V; sheath gas flow, 40 arb. units; aux gas flow, 5 arb. units; aux. gas heater temperature, 50°C. The HRMS/MS spectra (35.000 resolution) were recorded for the most intense three peaks, keeping a 10-s exclusive window. Stepped normalized collision energy was set at 40, 60, and 100. A Hypersil Gold UPLC C18 (2.1 × 100 mm, 1.9 μm) reversed phase column (Thermo Fisher Scientific, San Jose, CA, USA) was used for the separations. The mobile phase consisted of solvents A: ultra-pure H_2_O.1% (v/v) FA and B: ACN. A 16-min gradient method was used, varying as follows: 0–1 min, 5% B (isocratic gradient); 1–11 min, 5–95% B (linear gradient); 11–14 min, 95% B (isocratic gradient, column cleaning); 14–14.1 min, 95–5% B (linear gradient); 14.1–16 min, 5% B (isocratic, column equilibration). The flow rate was 260-ml min^−1^, and the injection volume was 5 μl. The column temperature was kept at 40°C, while the sample tray temperature was set at 10°C. All experiments were performed in triplicate.

#### Untargeted Metabolomics Data Processing

HRMS and MS/MS data were processed with Thermo Xcalibur and Compound Discoverer 3.1.1.12 (Thermo Fisher Scientific, CA, USA). For the untargeted metabolomics analysis, customizable processing workflow, including selection of spectra, RT alignment and signal correction, peak detection, and grouping and annotation of compounds, was applied to all the data set and described as follows: After the selection of imported raw files, retention time (RT) alignment was performed from 1 to 12 min as an upper limit with a maximum time shift alignment set at 2 min with 10-ppm mass tolerance. The peak-picking procedure was conducted on a full HRMS data scan between 0 and 1,100 Da, adopting criteria, such as minimum peak intensity (1,000,000), mass tolerance set up at 5 ppm but also by integrating selected isotopes and adducts. Finally, the straight elucidation and the annotation of compounds were performed according to the accurate mass, the potential adducts, and isotopes in correlation with MS/MS fragmentation spectra by comparing with commercial and noncommercial exported or implemented databases (e.g., Dictionary of Natural Products, library mzCloud, Chemspider, Masslist) as well as data from literature.

### Statistical Analysis

In both experiments, the TFW of plants was subjected to a mixed model for repeated measurements fit by restricted maximum likelihood (REML) estimation. “ID plants” were regarded as a random factor while “time” of sampling (T0 and T1- Exp.1 and T0 and T4 - Exp. 2) and “treatments” (NM and M) as fixed factors. Similarly, AMF colonization parameters (TC% and AC%) were subjected to the mixed model, as described above, with “ID plants” as a random factor and “time” as a fixed factor. For all parameters, normal distribution of residuals variance and normality was checked before analyses.

In the second experiment, the concentrations of Pi and ${\text{NO}}_{3}^{-}$ in the nutrient solutions were analyzed with a mixed model for repeated measurements, where “ID plants” were regarded as a random factor and “time” of sampling (T1, T2, T3, and T4) and “treatments” (M and NM) as fixed factors. To fulfill the assumptions of normality and homoscedasticity, Pi concentration from two NM plants was excluded from the statistical analysis. When the interaction between the fixed factors was not significant, a pairwise comparison test (*p* < 0.05) was computed on each significant principal effect (“time” and/or “treatments” factors). Data analyses were performed by IBM SPSS Statistics for Windows, version 26 (IBM Corp., Armonk, N.Y., USA).

The interpretation of metabolomics HRMS data involved a multivariate data analysis. The preprocessed datasets remaining after filtering criteria were imported into SIMCA 14.1 software (Soft Independent Modeling of Class Analogy; Umetric, Malmo, Sweden) for the assignment of the metabolic changes between M and NM plants at the end of both experiments. Principal component analyses (PCA), as well as the partial least squares discriminant analyses (PLS-DA), were performed according to Pareto correlation, while UV scaling was applied for the interpretation of clustering results between nutrient solutions of M and NM treatments at the end of the second experiment. To exclude the overfitting of the aforementioned PLS-DA models, a permutation test with *n* = 100 was performed. The discriminant variables were highlighted through Volcano-plots analyses generated with GraphPad Prism 7 (GraphPad Software, California, USA) and Compound Discoverer 3.1.1.12 (Thermo Fisher Scientific, CA, USA) on the basis of criteria such as *p* < 0.05 and *fold* change (FC) > 1.5. The dereplication and the matching of differential compounds were performed as described above.

## Results

### The Metabolite Profile of *A. officinalis* Associated With *R. irregularis* in a 9-Day Experiment Conducted in an S-H Cultivation System (Experiment 1)

#### Plant Total Fresh Weight and Root Colonization by AMF

The TFW of the M and NM plants was evaluated at T0 and T1. No significant interaction (*p* = 0.167) was reported between the two fixed factors “treatments” and “time”. Conversely, a significant effect was noticed for the fixed factor “treatments” (*p* < 0.001), as well as “time” (*p* = 0.004) separately. A significantly higher TFW of M plants was found as compared with NM plants. Moreover, the overall TFW of the two plant groups (M and NM) increased significantly between T0 and T1 (Table 1).

**Table 1:** Total fresh weight (TFW) of *A. officinalis* inoculated (M) or not (NM) with *R. irregularis* MUCL 41833 before (T0) and after 1 (T1) and 4 (T4) weeks of growth in the S-H cultivation system.

**Treatments**	**Exp. 1**	**Exp. 2**	**Time**	**Exp. 1**	**Exp. 2**
NM	2.3 ± 0.7 a	7 ± 1 a	T0	3.7 ± 0.5^*^	6 ± 0.8^*^
M	5.8 ± 0.7 b	9.6 ± 1 a	T1	4.4 ± 0.5^*^	–
			T4	–	10.6 ± 0.8^*^

The parameters are expressed as mean ± standard errors (SE) of 7 M and 6 NM (Exp. 1) and 7 M and 7 NM (Exp. 2) replicates. Values within the same column followed by different lowercase letters, for fixed factor “treatment,” and by ^*^, for fixed factor “time”, are significantly different according to the mixed model for repeated measurements (p < 0.05).

Root colonization of the M and NM plants was evaluated prior to their transfer in the containers (T0) and at the end of the experiment (i.e., after 9 days of growth in the S-H cultivation system – T1). A significant effect (*p* < 0.05) of the fixed factor “time” was noticed for TC% and AC%. Both parameters significantly decreased between the start and the end of the experiment. No root colonization was observed for the NM plants (Table 2).

**Table 2:** Total colonization (TC%) and arbuscules colonization (AC%) of *A. officinalis* inoculated (M) with *R. irregularis* MUCL 41833 before (T0) and after 1 (T1) and 4 (T4) weeks of growth in the S-H cultivation system.

**Fixed factor**	**Exp. 1**		**Exp. 2**	
**Time**	**TC**	**AC**	**TC**	**AC**
T0	62 ± 2^*^	35 ± 2^*^	57 ± 2^*^	26 ± 2
T1	55 ± 2^*^	18 ± 2^*^	–	–
T4	–	–	50 ± 2^*^	24 ± 2

The parameters are expressed as mean ± standard errors (SE) of 7 M replicates. Values within the same column followed by ^*^ are significantly different according to the mixed model for repeated measurements (p < 0.05).

#### Metabolite Profiles of M and NM *A. officinalis* Plants and of Nutrient Solution

**Figure 2:**
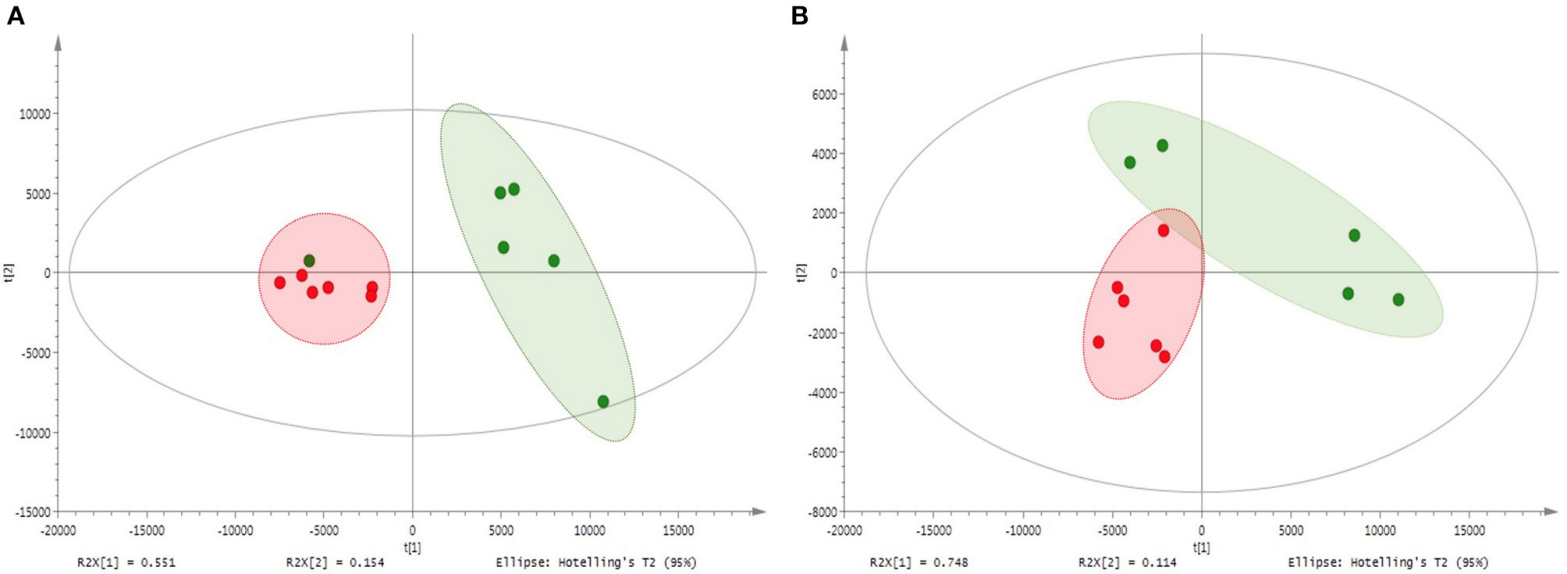
Principal component analysis (PCA) – comparison of UHPLC-HRMS metabolic profiles from M and NM root **(A)** and shoot **(B)** samples after 1 week (T1) of growth in the S-H cultivation system (M_T1: green dots; NM_T1: red dots).

The metabolite profiles of shoots and roots of M and NM plants as well as of nutrient solutions were assessed *via* UHPLC-HRMS mass spectrometry. Sample analyses revealed 220 and 420 different mass signals for shoot and root samples, respectively, and the unsupervised PCA with Pareto correlation revealed a clear contrast and discrimination between M and NM plants for both plant parts (Figure 2). Interestingly, root and shoot parts showed a similar clustering pattern without any overlapping between M and NM individuals from the same dataset. Furthermore, higher proximity and similitude of individuals were observed for the NM plants in both shoots and roots, in comparison to the M plants, which were found more widespread in the model. Curiously, the root metabolic profile of one NM plant was closer to the M plants than to the NM plants (Figure 2A), while the corresponding shoot part was considered as an outlier and was removed from the PCA model (Figure 2B). Preliminary PCA analyses showed that AMF colonization significantly affected plant metabolism in both root and shoot samples. The partial least squares-discriminant analysis of M and NM shoots and roots confirmed the results obtained by PCA, showing clear differentiation between M and NM samples. The statistical significance test obtained from 100 possible rearrangements of data points confirmed the aforementioned model (Supplementary Figure 1).

**Figure 3:**
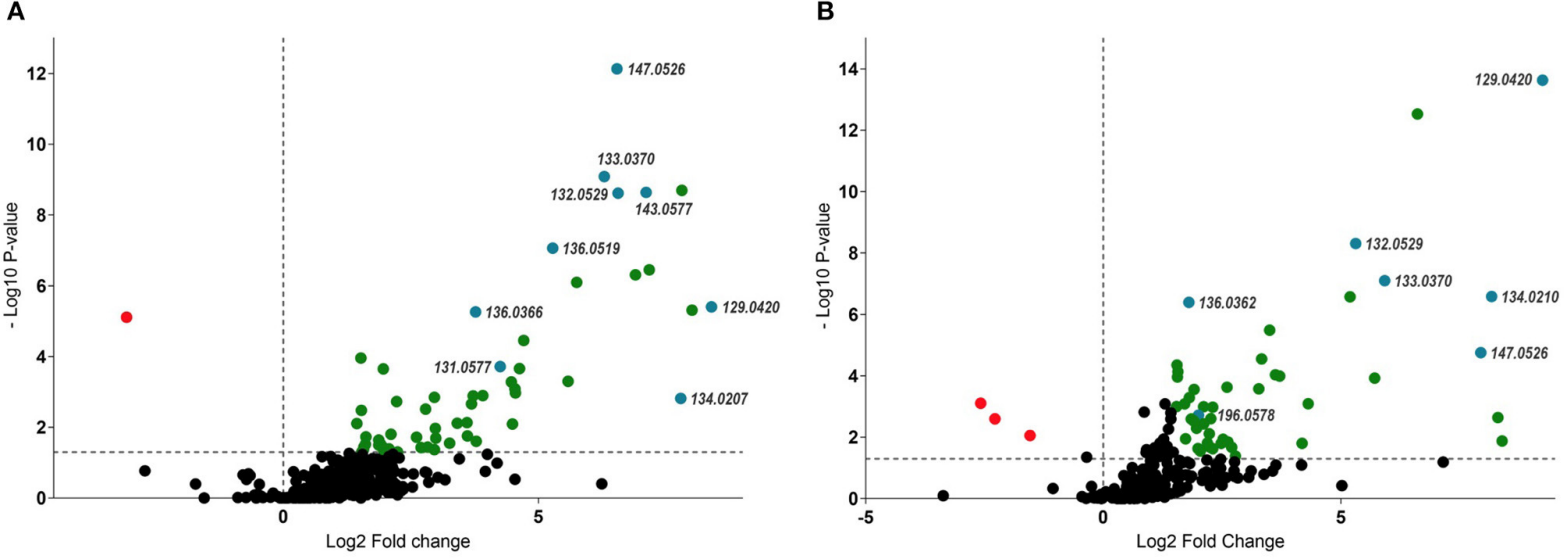
Volcano-plot analysis – identification of up and downregulated compounds (*p* < 0.05 and FC > 1.5) between M and NM root **(A)** and shoot **(B)** samples after 1 week (T1) of growth in the S-H system. Statistically significant upregulated SMs were represented in green, while PMs were highlighted in blue (right side of the plots). Downregulated compounds were represented in red (left side of the plots). Numbers represent pseudomolecular ions (*m/z*).

Variables of importance were highlighted in the volcano-plot analyses obtained with specific data-filtering criteria such as *p* < 0.05 and FC > 1.5. Among the 220 and 420 mass signals identified for shoot and root samples of M and NM plants, 55 and 49, respectively, passed the defined threshold (Figures 3A,B). Afterwards, those compounds were tentatively identified by matching the corresponding accurate mass and MS/MS spectra pattern with already published data from literature and compound libraries (see Section Dereplication of PMs and SMs).

**Figure 4:**
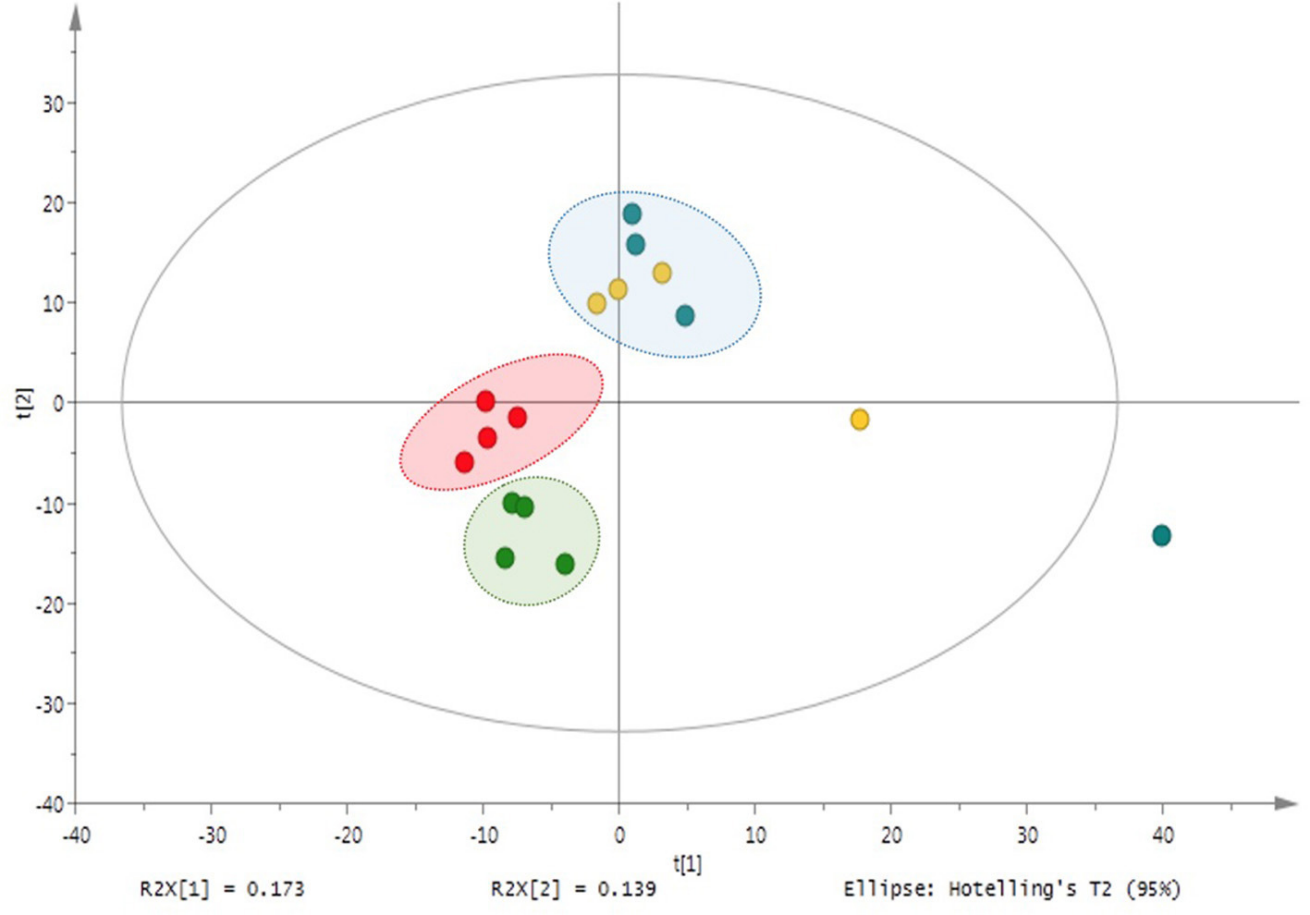
Principal component analysis (PCA) of M and NM nutrient solutions –UHPLC-HRMS-based metabolite variation after 1 week (M_T1: blue dots; NM_T1: yellow dots) and 4 weeks (M_T4: green dots; NM_T4: red dots) of plant growth in the S-H cultivation system.

In contrast to plant samples, PCA and PLS-DA analyses of the nutrient solutions from M and NM plants did not follow the same clustering pattern as in shoots and roots. In particular, no significant differences in mass signals were underlined in the different HRMS-(-) ESI profiles, meaning that the association of plants with the AMF did not affect the root exudation and the release of compounds in the nutrient solution within a period of 9 days. As a result, metabolic profiles observed in nutrient solutions of M and NM plants were represented in a single cluster in the plot (Figure 4). Volcano-plot analysis of post-processing HRMS data confirmed PCA and PLS-DA models. In fact, despite the higher number of mass signals (>500) detected in the nutrient solution of M and NM plants, no significant difference was observed according to the same data-filtering criteria (*p* < 0.05 and FC > 1.5).

### The Metabolites Profile of *A. officinalis* Associated With *R. irregularis* in a 30-Day Experiment Conducted in an S-H Cultivation System (Experiment 2)

#### Plant Total Fresh Weight and Root Colonization by AMF

The total fresh weight of the M and NM plants was measured at T0 and T4. No significant interaction (*p* = 0.926) was reported between the two fixed factors “treatments” and “time”, while only a significant effect was found for the fixed factor “time” (*p* < 0.001). The overall TFW of the two groups (M and NM) of plants increased between T0 and T4 (Table 1).

Root colonization of the M and NM plants was evaluated prior to the transfer in the containers (T0) and at the end of the experiment (i.e., after 4 weeks of growth in the S-H cultivation system – T4). A significant effect of the fixed factor “time” was reported only for TC% (*P* = 0.032), with a decrease between the start and the end of the experiment. The AC% remained stable during the experiment. No root colonization was observed for the NM plants (Table 2).

#### Minerals Depletion in Hoagland^dil100X^ Low-P Solution

The concentration of Pi and ${\text{NO}}_{3}^{-}$ in the bottles of the M and NM plants followed a similar decrease over time (Supplementary Figure 2). The depletion of both minerals in the circulating solution resulted in a concomitant uptake/immobilization by the plant-AMF associates.

No significant interactions (*p* = 0.704 and *p* = 0.866) were noticed between the fixed factors “treatments” and “time” for both Pi and ${\text{NO}}_{3}^{-}$. Conversely, a significant effect (*p* < 0.001) of the fixed factor “time” was noticed. According to the pairwise comparison test (*p* < 0.05), a significant difference in the Pi and ${\text{NO}}_{3}^{-}$ concentration, represented as the average of M and NM plants at the different time points, was noticed at T4 as compared with the other time samplings. A decrease by 88 and 35% of [Pi] and [${\text{NO}}_{3}^{-}$], respectively, was noticed at the end of the experiment.

#### Metabolite Profiles of M and NM *A. officinalis* Plants and of Nutrient Solution

**Figure 5:**
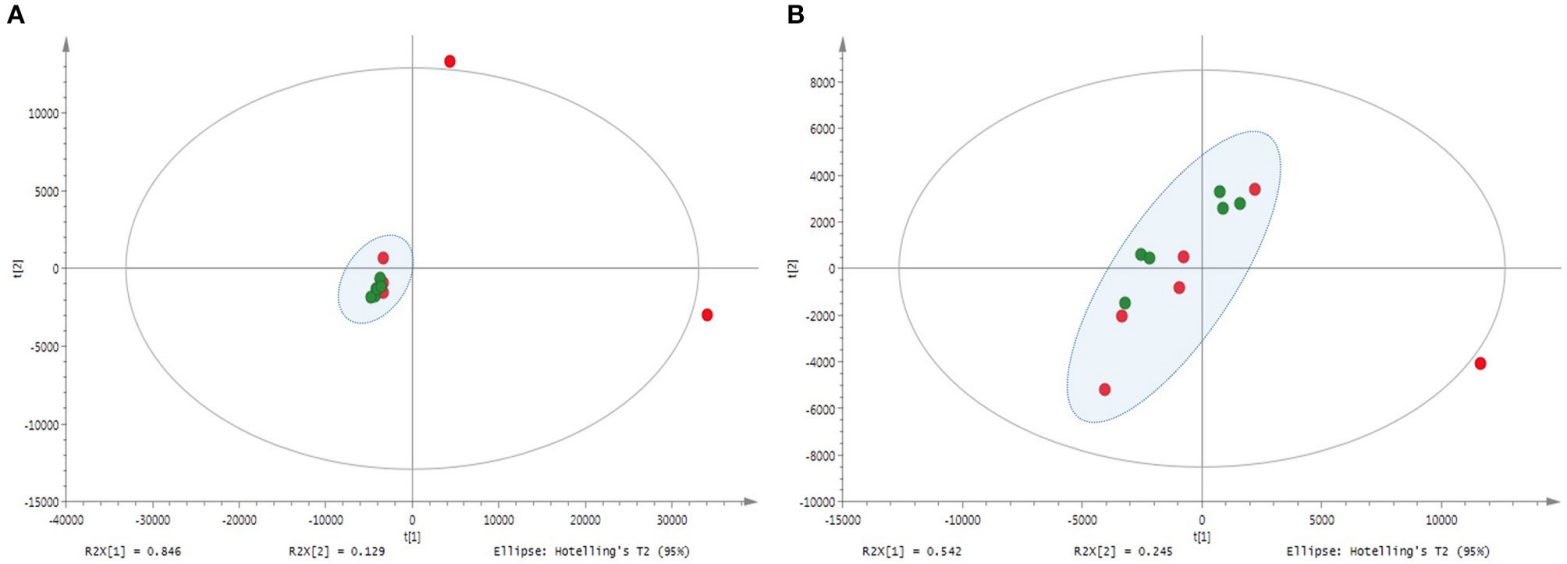
Principal component analysis (PCA) – comparison of UHPLC-HRMS metabolic profiles from M and NM root **(A)** and shoot **(B)** samples after 4 weeks (T4) of growth in the S-H cultivation system (M_T4: green dots; NM_T4: red dots).

The metabolite profiles of shoots and roots of M and NM plants, as well as of nutrient solutions, were assessed *via* UHPLC-HRMS. Clustered analysis was illustrated through an unsupervised PCA model (Figure 5) with Pareto correlation and confirmed by PLS-DA analysis (Supplementary Figure 3). Shoot and root samples of M and NM plants were clustered as a single group in the plot, and no significant differences in mass signals were observed between metabolic profiles of M and NM plants. However, roots of M and NM plants were closely clustered (Figure 5A), with the exception of 2 NM individuals plotted at the upper limits of the model. Conversely, shoot samples from both M and NM plants were widespread in the model, reflecting the higher metabolic variability in the shoots, independent of AMF colonization (Figure 5B).

Regarding the metabolic analysis of the nutrient solutions from M and NM treatments at the end of the experiment (T4), more than 600 mass signals were detected. Interestingly, distinguishable clustering between nutrient solutions of M and NM plants was noticed through an unsupervised UV-scaling PCA analysis (Figure 4). Results were confirmed by PLS-DA analysis (Supplementary Figure 4). UHPLC-HRMS data were further interpreted by Volcano-plot analysis (Supplementary Figure 5), with the same criteria as described earlier (*p* < 0.05 and FC > 1.5). Two compounds with *m*/*z* 206.0570 and *m*/*z* 334.1049 were significantly induced in the nutrient solutions of the M plants. A tentative annotation was proposed by matching the corresponding accurate mass and MS/MS spectra pattern with already published data from literature and compound libraries (see Section Dereplication of PMs and SMs).

Besides the AMF key role in the induction of specific compounds, the nutrient solutions at T1 and T4 significantly differed in the abundance of exuded metabolites. Among them, 37 compounds were mostly accumulated at T1, while 45 metabolites were induced at T4 (*p* < 0.05 and FC > 1.5) (Supplementary Figure 6). Results of Volcano-plot analysis suggested that the PMs and SMs exudation rate significantly changed according to the time spent by the plants in the S-H culture system. However, for most of them, it was not related to the AMF colonization.

### Dereplication of PMs and SMs

The untargeted metabolic analysis of M and NM shoots, roots, and nutrient solutions of both experiments revealed the up and downregulation of several different mass signals. The dereplication process was performed with commercial and noncommercial databases and by comparison with available literature data, leading to the identification of 36 upregulated and downregulated fragments tentatively assigned to PMs and SMs.

Ten compounds from the primary metabolism were tentatively identified according to their [M-H]^−^ pseudomolecular ions and by their full MS/MS fragmentation patterns (Table 3a). Six amino acids, among which were aspartic and glutamic acids, were significantly increased, especially in M root parts, and four organics acids, tentatively annotated as threonic acid, malic acid, gluconic acid, and phenylacetic acid, were only identified in shoots of M plants. SMs production in shoots and roots was also modulated by AMF (Table 3b). Twenty-six different compounds, belonging to different chemical groups, were tentatively characterized. Among them, 16 peaks were annotated as C_6_-C_3_ mono-, di-, tri, and tetramers derivatives of caffeic acid, two compounds as C_6_-C_1_ benzoic acid derivatives, and four compounds were characterized as triterpenes saponins according to the [M-H]^−^ pseudomolecular ion and their corresponding MS/MS fragments.

**Table 3:** Up and downregulated **(a)** PMs and **(b)** SMs in shoots and roots of plants inoculated with *R. irregularis* after 1 week of growth in the S-H cultivation system (Exp. 1).

		**Peak**	**Proposed phytochemicals**	**R**_**t**_ **(min)**	**Precursor Ion - [M-H]^**−**^**	***m/z* calcd**.	**Δm (*ppm*)**	**MS/MS Fragment ions (*m/z*)**	**Chemical formula**	**Distribution** ^**a**^	**References**
S and R metabolites content^b^	(**a**) Primary metabolism	1	Asparagine	1.44	131.0453	132.0529	−7.3	131, 115, 88, 71	C_4_H_8_N_2_O_3_	S (5.19), R (6.56)^↑^	Zengin et al., 2018
		2	L-Aspartic acid	1.46	132.0294	133.0370	−6.6	132, 115, 88, 71	C_4_H_7_NO_4_	S (2.19), R (6.29)^↑^	Brieudes et al., 2016
		3	Glutamic acid	1.44	146.0451	147.0526	−5.3	146, 128, 102	C_5_H_8_NO_4_	S (5.31), R (6.54)^↑^	Brieudes et al., 2016
		4	Gluconic acid	1.49	195.0505	196.0578	−2.6	177, 160, 129, 87, 75	C_6_H_12_O_7_	S (1.96)^↑^	Brieudes et al., 2016
		5	L-Threonic acid	1.54	135.0290	136.0366	−6.8	135, 117, 89, 75, 61	C_4_H_8_O_5_	S (6.61), R (3.77)^↑^	Brieudes et al., 2016
		6	DL-Malic acid	1.59	133.0134	134.0210	−6.6	133, 115, 89, 72, 71	C_4_H_6_O_5_	S (8.17), R (7.79)^↑^	Li et al., 2004
		7	DL-pyroglutamic acid	1.65	128.0344	129.0420	−7.1	128, 82, 62	C_5_H_7_NO_3_	S (9.24), R (8.39)^↑^	Shi et al., 2020
		8	N-Acetylalanine	2.14	130.0501	131.0577	−6.7	130, 88	C_5_H_9_NO_3_	R (4.25)^↑^	–
		9	Methylpyroglutamate	2.23	142.0502	143.0577	−5.5	142, 100, 98, 58	C_6_H_9_NO_3_	R (7.11)^↑^	–
		10	Phenylacetic acid	3.65	135.0443	136.0519	−6.2	93, 72	C_8_H_8_O_2_	R (5.28)^↑^	Lee et al., 2017
	(**b**) Secondary metabolism	11	Syringic acid	3.71	197.0453	198.0523	−1.3	179, 151, 135, 123, 72	C_9_H_10_O_5_	R (4.55)^↑^	Taamalli et al., 2015
		12	*p*-Hydroxybenzoic acid	5.03	137.0236	138.0311	−6.3	135, 93, 65	C_7_H_6_O_3_	R (6.90)^↑^	Gómez-García et al., 2021
		13	Chorismic acid	5.24	225.0404	226.0472	−0.9	207, 179, 137, 109, 61	C_10_H_10_O_6_	R (2.70)^↑^	Khera et al., 2017
		14	Caffeic acid	5.39	179.0344	180.0417	−3.3	135, 109, 89, 73	C_9_H_8_O_4_	S (2.11)^↑^	Gómez-García et al., 2021
		15	Methyl syringinoside	5.25	547.2039	548.2099	1.3	191, 176, 161, 121, 93, 71	C_24_H_36_O_14_	R (1.54)^↑^	Srinroch et al., 2020
		16	Lithospermic acid	5.81	537.1050	538.1106	2.11	339, 295, 269, 197, 179, 161, 135, 109, 73	C_27_H_22_O_12_	S (2.28), R (3.07)^↓^	Liu et al., 2007
		17	Anchusoside-9	6.07	827.4449	828.4502	1.8	665, 503, 161, 113, 85, 71	C_42_H_68_O_16_	S (2.25)^↑^	Romussi et al., 1984
		18	Methyl syringin	6.40	385.1510	386.1571	1.5	223, 191, 176, 161, 121, 93, 71	C_18_H_26_O_9_	R (1.52)^↑^	Park et al., 2017
		19	Rosmarinic acid (RA)	6.55	359.0777	360.0840	1.1	197, 179, 161, 135, 123, 73, 62	C_18_H_16_O_8_	R (2.11)^↑^	Krzyzanowska-Kowalczyk et al., 2018
		20	Salvianolic acid C	6.86	491.0992	492.1051	1.6	311, 267, 197, 185, 179, 135, 109, 73	C_26_H_20_O_10_	S (5.71)^↑^	Finimundy et al., 2020
		21	Syringin	6.89	371.1353	372.1415	1.3	176, 161, 121	C_17_H_24_O_9_	S (2.04)^↑^	Filipek et al., 2019
		22	Ferulic acid	7.04	193.0502	194.0574	−2.2	179, 161, 133	C_10_H_10_O_4_	S (3.33), R (2.22)^↑^	Gómez-García et al., 2021
		23	Methyl lithospermic acid	7.11	551.1202	552.1262	1.5	339, 321, 293, 231, 185, 179, 161, 135, 109, 73	C_28_H_24_O_12_	R (2.79)^↑^	Liu et al., 2007
		24	Dehydro SA B	7.12	715.1324	716.1372	2.7	357, 339, 295, 185, 135, 109, 72	C_36_H_28_O_16_	S (4.31), R (4.47)^↑^	Li et al., 2018
		25	Methyl rosmarinic acid	7.17	373.0934	374.0996	1.5	197, 179, 160, 135, 123, 73	C_19_H_18_O_8_	S (1.99)^↑^	Krzyzanowska-Kowalczyk et al., 2018
		26	8-hydroxy-9”-methyl dehydro SA B	7.24	745.1430	746.1478	2.7	467, 387, 339, 295, 193, 185, 151, 133, 109, 73	C_37_H_30_O_17_	R (7.17)^↑^	Liu et al., 2007
		27	8-hydroxy-9”-methyl SA B	7.34	747.1588	748.1634	2.6	467, 389, 339, 295, 195, 185, 151, 135, 109, 73	C_37_H_32_O_17_	R (3.41)^↑^	Liu et al., 2007
		28	Salvianolic acid B (SA B)	7.47	717.1479	718.1528	2.2	357, 339, 321, 295, 265, 197, 185, 161, 135, 109, 73	C_36_H_30_O_16_	R (3.72)^↑^	Wu et al., 2006
		29	8-hydroxy-9'-methyl dehydroRA	7.50	387.0731	388.0789	2.4	207, 179, 135, 121, 109	C_19_H_16_O_9_	R (4.54)^↑^	Grzegorczyk-Karolak et al., 2019
		30	Isobavachalcone hexoside	7.92	485.1827	486.1884	2.0	177, 163, 145, 117	C_26_H_30_O_9_	R (5.58)^↑^	Cioffi et al., 2003
		31	Salvianolic acid F	7.93	313.0723	314.0793	1.6	203, 179, 161, 133, 123	C_17_H_14_O_6_	S (2.48)^↑^	Grzegorczyk-Karolak et al., 2019
		32	Oleanolic acid triglycoside	8.06	939.3993	940.5026	3.6	808, 617, 455, 159, 129, 111, 87, 71	C_48_H_78_O_18_	S (2.26)^↑^	-
		33	Anchusoside-1	8.44	779.4612	780.4654	3.1	617, 455, 141, 112, 71	C_43_H_70_O_15_	S (1.73)^↑^	Romussi et al., 1979
		34	Anchusoside-2	9.25	941.5106	942.5183	−0.9	779, 617, 455, 161, 113, 85, 71	C_48_H_78_O_18_	S (1.91)^↑^	Romussi et al., 1979
NS^c^		35	Scoparone	6.25	205.0497	206.0574	−4.4	177, 161, 143, 133, 119	C_11_H_10_O_4_	NS (3.21)^↑^	Wang et al., 2007
		36	Byakangelicin	10.89	333.0976	334.1047	−1.0	303, 249, 202, 147, 131, 125	C_17_H_18_O_7_	NS (9.5)^↑^	Zhang et al., 2009

*Upregulated SMs (exudates) in nutrients solutions (NS) of M plant after 4 weeks (T4) of growth in the S-H cultivation system*.

a *Numbers with a parenthesis represent the fold change of each compound in “M vs. NM” plants and corresponding nutrient solutions. Green arrows indicate the upregulation while red arrows the downregulation in shoot (S), roots (R), and/or nutrient solutions (NS) of M plants*.

b *PMs and SMs affected by the AMF-plant symbiosis in shoot and root parts of the S-H cultivation system at T1 of the experiment*.

c *SMs affected by the AMF-plant symbiosis in nutrient solutions (exudates) of the S-H cultivation system at T4 of the experiment*.

*Rt, retention time; Δm, mass errors; [M-H]-, m/z of the pseudomolecular ion in negative and positive ionization modes, respectively; m/z calcd, theoretical m/z value*.

In addition, compound 26 was found to be the most upregulated metabolite from the AMF symbiosis. This compound presented a pseudo molecular ion at *m*/*z* 745.1430 [M-H]^−^ and shared a similar MS/MS fragmentation pattern with compound 24, tentatively assigned as dehydro-SA B. Compound 24 has been previously identified by Liu et al. (2007) by the extensive use of MS and MS/MS spectra. It shows a pseudo molecular ion at *m*/*z* 715.1324 [M-H]^−^ and prominent MS/MS fragment ions at *m*/*z* 339, 295, and 185, confirming the tetrameric C_6_-C_3_ configuration as well as ions at *m/z* 161, 135, 121, 109, and 73 derived from further cleavage of the single C_6_-C_3_ units (C_9_H_10_O_5_). Indeed, the MS/MS spectra of compound 24 show ions at *m*/*z* 339, resulting from the neutral loss of a dehydroxy-danshensu (C_9_H_10_O_4_) and of a hydroxy-caffeic acid (C_9_H_8_O_5_), while the fragment ions at *m*/*z* 295 and 185 emerged from a further loss of a CO_2_ and of a benzendiol (-109 Da) unit, respectively. This clearly suggested the presence of a hydroxybenzofuran ring in the structure (Table 4). A similar fragmentation pattern has been found for compound 26. However, the mass difference of 30 Da with respect to compound 24 was assigned, based on the MS/MS data, to the presence of an additional methyl (-CH_3_) and of a hydroxy group (-OH) at positions 9” and 8, respectively. In particular, the diagnostic MS/MS fragments at *m*/*z* 467, 193, and 151, not present in the MS/MS spectra of compound 24, suggested the methyl-caffeic unit (CH_3_ at position O-9”) and the addition of the –OH group at C-8 (Liu et al., 2007) (Table 4). According to these findings, compound 26 was tentatively identified as 8-hydroxy-9”methyl-dehydro-SA B and annotated with a chemical formula of C_37_H_30_O_17_. Similarly, compound 27 was tentatively assigned as an SA B derivative. It showed a pseudomolecular ion at *m/z* 747.1588 [M-H]^−^ with a chemical formula of C_37_H_32_O_17_ corresponding to that of compounds 26 with the absence of a double bond at position 7”. Therefore, the structure was tentatively assigned as 8-hydroxy-9”methyl-SA B.

**Table 4:** Chemical structures and the fragmentation pattern of compounds 18, 23, and 25–28 identified by ESI-HRMS and MS/MS analysis.

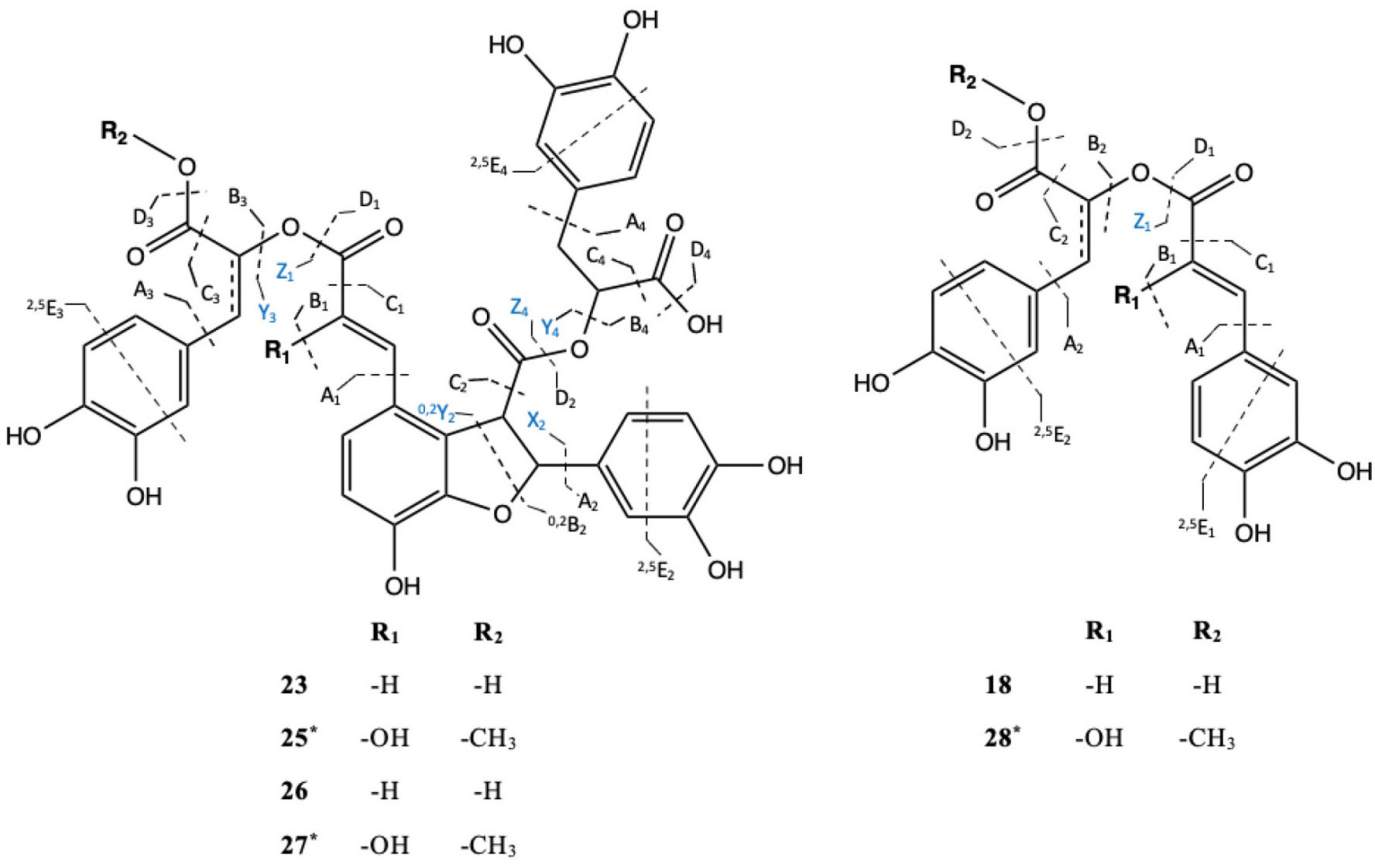		
Comp.	[M-H]^−^	MS/MS (*m/z*)
19	359.0777	197 (D_1_), 179 (B_1_), 161(B_1_/D_2_), 135 (B_1_/C_2_), 123, 73 (^2,5^E)
23	715.1324	357 (^0,2^Y_2_ or Y_3_/Y_4_), 339(D_1_/Y_4_), 321(D_1_/D_2_), 295(D_1_/C_2_), 197 (Z_4_), 185 (D_1_/X_2_/C_2_), 161(B/D), 133(B_1_/C_1_ or B_3_/C_3_), 135 (^0,2^B_2_/C_2_ or B_4_/C_4_), 109 (A), (^2,5^E)
25	745.1430	467 (A_1_), 387 (Y_2_), 339 (D_1_/D_2_), 295 (B_1_/D_1_/C_2_), 197 (Z_4_), 193 (B_3_ or ^0,2^Y_2_ /Y_3_), 185 (B_1_/D_1_/C_2_/A_2_), 151 (C_1_/^0,2^Y_2_), 133(B_1_/C_1_ or B_3_/C_3_), 135 (^0,2^B_2_/C_2_ or B_4_/C_4_), 109 (A), (^2,5^E)
26	747.1588	467 (A_1_), 389 (^0,2^Y_2_), 339 (D_1_/D_2_), 295 (B_1_/D_1_/C_2_), 197 (A), 195 (Z_4_), 185 (B_1_/D_1_/C_2_/A_2_), 151 (C_1_/^0,2^Y_2_), 133(B_1_/C_1_ or B_3_/C_3_), 135 (^0,2^B_2_/C_2_ or B_4_/C_4_), 109 (A), (^2,5^E)
27	717.1479	357 (Y_3_/Y_4_), 339(D_1_/Y_4_), 321(D_1_/D_2_), 295(D_1_/C_2_), 197 (Z_1_ or Z_4_), 185 (D_1_/X_2_/C_2_), 161(B/D), 135 (^0,2^B_2_/C_2_ or B_3_/C_3_ or B_4_/C_4_), 109 (A), 73 (^2,5^E)
28	387.0731	207 (Z_1_), 179 (D_2_), 161 (B_1_/D_1_), 135 (B_1_/C_1_ or^0,2^B_2_/C_2_), 109 (A)

^*^Compounds possessing a double bond at C7' or C7”.

Among the induced ions, dimeric C_6_-C_3_ forms were identified. Compound 29 presented a pseudomolecular ion at *m*/*z* 387.0731 [M-H]^−^ with a prominent MS/MS fragment at *m/z* 135 [M-H]^−^, suggesting the presence of another dimeric form of caffeic acid units. Interestingly, its pseudomolecular ion corresponded to one of the MS/MS fragments, resulting from the cleavage of compound 25. Thus, a common part between those two molecules was deduced. Our suggestion was further confirmed by the presence of the diagnostic MS/MS fragments at *m*/*z* 207, 179, 135, and 109, corresponding to cleavage of the methyl-caffeoyl and hydroxy caffeoyl units as shown in Table 4. Therefore, compound 29 was tentatively assigned as 8-hydroxyl-9'-methyl dehydro RA with a chemical formula of C_19_H_16_O_9._

On the other hand, compound 16 represents the only downregulated SM in M plants in both shoot and root tissues, respectively. It showed a pseudomolecular [M-H]^−^ ion at *m*/*z* 537.1050 and MS/MS ion fragments at *m*/*z* 339 and 295, as well as major ions fragments at *m*/*z* 179, 161, 135, and 109, corresponding to the characteristic cleavage of the three C_6_-C_3_ units of lithospermic acid (C_27_H_22_O_12_). In addition, compound 16 showed a mass difference of −14 Da with respect to compound 23, whose structure was assigned as the methylated derivative of lithospermic acid at position 9”.

Arbuscular mycorrhizal fungi symbiosis affected the expression of four glycosylated triterpernoids. In our analysis, compounds 17, 32, 33, and 34 were annotated as saponins and showed a strong accumulation in shoots of M plants. In addition, compound 17 shows a molecular ion at *m*/*z* 828.4502 [M-H]^−^, tentatively assigned to the oleanolic acid glycoside Anchusoside-9 through the two characteristic MS/MS fragments at *m*/*z* 665 and 503. This resulted from the consecutive neutral loss of two hexose units from the structure (−162 and −324 Da) (Romussi et al., 1984). Compounds 32-34 shared a common fragment ion at *m*/*z* 455, corresponding to the aglycone part of the structure (oleanolic acid), confirming their structural similitudes. Specifically, compounds 32 and 33 were tentatively characterized as tri- and di-glycosidic esters of oleanolic acid, respectively. They both shared a prominent characteristic MS/MS ion at *m*/*z* 617, corresponding to a common glucopyranosyl oleanolate unit. Moreover, compound 32 presents additional fragment ions at *m*/*z* 808 [M–H−133]^−^, resulting from the neutral loss of one pentose and at *m*/*z* 323, corresponding to the cleavage of a disaccharide unit that contained the abovementioned pentose and a glucuronic acid methyl ester moiety. Based on these findings and according to the pseudomolecular ion [M-H]^−^ at *m*/*z* 939.3993, compound 31 was tentatively assigned as a tri-glycosylated oleanolic acid methyl ester derivative, detected for the first time in *A. officinalis*. Conversely, compound 33, with a molecular ion at *m*/*z* 779.4612 and the abovementioned characteristic MS/MS fragment, was tentatively assigned as the di-hexoside oleanolic acid derivative Anchusoside-1, commonly found in *A. officinalis*. Compound 34, showed a pseudomolecular ion at *m*/*z* 941.5106 [M-H]^−^ and was tentatively assigned as the trihexoside oleanolic acid ester Anchusoside-2. Based on the main MS/MS fragment at *m*/*z* 455, corresponding to oleanolic acid, we assumed the presence of a trihexoside unit in the structure. This was further confirmed by the MS/MS fragments at *m*/*z* 779 and 617, corresponding to the successive loss of three glucosyl moieties (−162, −324, and −486 Da).

Regarding the nutrient solutions, two accumulated compounds were detected in the M plants of Exp. 2 (T4). A potential chemical formula of C_11_H_10_O_4_ and C_17_H_18_O_7_ was determined according to the [M-H]^−^ pseudomolecular ion at *m*/*z* 205.0497 and *m*/*z* 333.0976, respectively, leading to the tentative identification, according to their MS/MS fragmentation pattern, of the coumarin scoparone and of the furanocoumarin byakangelicin. Scoparone showed characteristic MS/MS ion fragments at *m*/*z* 161 and 133, related to the successive loss of -CO_2_ from the lactone ring and of –OCH_3_, respectively, while the ion fragment at *m/z* 119 corresponded to the loss of a further methyl radical (Concannon et al., 2000; Wang et al., 2007). Byakangelicin presented MS/MS ion fragments at *m*/*z* 303 (C_16_H_15_O_6_)^−^ and at *m*/*z* 290 (C_16_H_18_O_5_)^−^ emerged from the loss of –OCH_3_ and –CO_2_, respectively, of the lactone ring. Further rearrangements of the structure, for the formation of a more stable ion, gave the fragment ions at *m*/*z* 203 related to the loss of the side chain and of a carbonyl, while fragments at *m*/*z* 147 and 131, generated from the consecutive loss of –CO_2_ and of other carbonyl units, further confirmed the structure of compound 36 (Zhang et al., 2009).

## Discussion

Plants interact with a complex community of beneficial microorganisms developing in seeds, leaves, and roots, providing nutritional assistance, stimulating growth, and inducing resistance against pest and diseases as well as against abiotic stresses. Colonization of plant tissues often induces quantitative and qualitative changes in PMs and SMs; some of which may be of commercial interest. Thus, a targeted approach with selected microbial inoculants applied under highly controlled conditions may be of interest to increase or stimulate the production of specific high-value metabolites. AMF are among those microorganisms improving plant nutrition and growth, stimulating defense mechanisms (Chen et al., 2018), securing plant development under abiotic stress conditions (Plouznikoff et al., 2016), and impacting the up and downregulation of specific PMs (Pedone-Bonfim et al., 2015) and SMs (Zeng et al., 2013) in plants. Here, two successive experiments were conducted under an S-H cultivation system to investigate the role of AMF in PMs and SMs production in shoots, roots, and exudates of the medicinal plant *A. officinalis*. An untargeted UHPLC-HRMS metabolomics approach combined with multivariate data analysis enabled to provide a broad picture of the *A. officinalis* metabolic profile changes as a result of AMF colonization.

### Adequacy of the S-H Cultivation System for PMs and SMs Analysis in AMF-Colonized Plants

To evaluate the adequacy of the S-H cultivation system, plant and AMF parameters were assessed. *Anchusa officinalis* growth was clearly observed in both experiments, in the presence and absence of AMF. This was evidenced by an increase in TFW between the start and the end of both experiments and by depletion of Pi and ${\text{NO}}_{3}^{-}$ in the nutrient solutions (monitored in Exp. 2). However, significant differences in TFW were noticed between M and NM plants only in Exp. 1, with higher values recorded in the AMF-colonized plants. This could be attributed to the higher TFW of M plants at transfer from pots to the S-H system. Moreover, the plants transfer may have caused an initial stress, which was probably better supported by the M plants, due to the presence of its fungal associate. However, no differences in TFW were noticed between the two treatments in Exp. 2. The plants were most probably adapted to the longer period of growth in the S-H system, and the initial stress, noticed in Exp.1, was no longer present at the end of this experiment (as reported below for AMF colonization assessment). Besides, each plant received strictly the same amount of nutrients flowing on the root system during several weeks, and similar minerals depletion was reported in the presence and in the absence of AMF, resulting in no visible impact on plant growth. Similar trends have been reported in other studies, using the S-H cultivation system (Garcés-Ruiz et al., 2017; Calonne-Salmon et al., 2018).

Root colonization in both experiments was high, even if significant decreases were noticed between the start and the end of experiments. This was particularly marked in Exp. 1 for TC% and AC%, while it was less noticeable in Exp. 2 with only TC%, showing a significant decrease. The decrease was possibly related to stress conditions occurring between transfer from pot to S-H system and final harvest, with extraradical mycelium partially damaged at plant transfer to the S-H system, requiring more time for the fungus to recover and extend in the root system. The differences were less noticeable in Exp. 2, in which the final observations were done 30 days after transfer in the S-H cultivation system, with a probable better recovery of the AMF. Numerous arbuscules were observed in both experiments with AC% close to 20 and above 20% at the end of Exp. 1 and 2, respectively, suggesting that the symbiosis was functional throughout the duration of experiments.

### Impact of AMF on PMs and SMs in Shoots, Roots, and Exudates of *A. officinalis* Grown in the S-H Cultivation System

Besides the phenotypic trait changes between M and NM plants, primary and secondary metabolism was significantly impacted in the presence of AMF. The untargeted metabolomics approach performed in Exp.1, after 9 days in the S-H cultivation system, showed an upregulation of in total 35 compounds between PMs and SMs, with *fold* change values ranking from 1.52 to 9.50, and a downregulation of one single SM with a *fold* change decrement of 2.28 and 3.07 in shoot and root tissues of M plants. Conversely, in Exp. 2, while no significant differences in PMs and SMs were assessed between M and NM plants, an upregulation of two compounds was reported in the exudates of M plants at T4, with *fold* change values of about 3.21 and 9.5.

#### Shoots and Roots

##### Impact on Primary Metabolism

**Figure 6:**
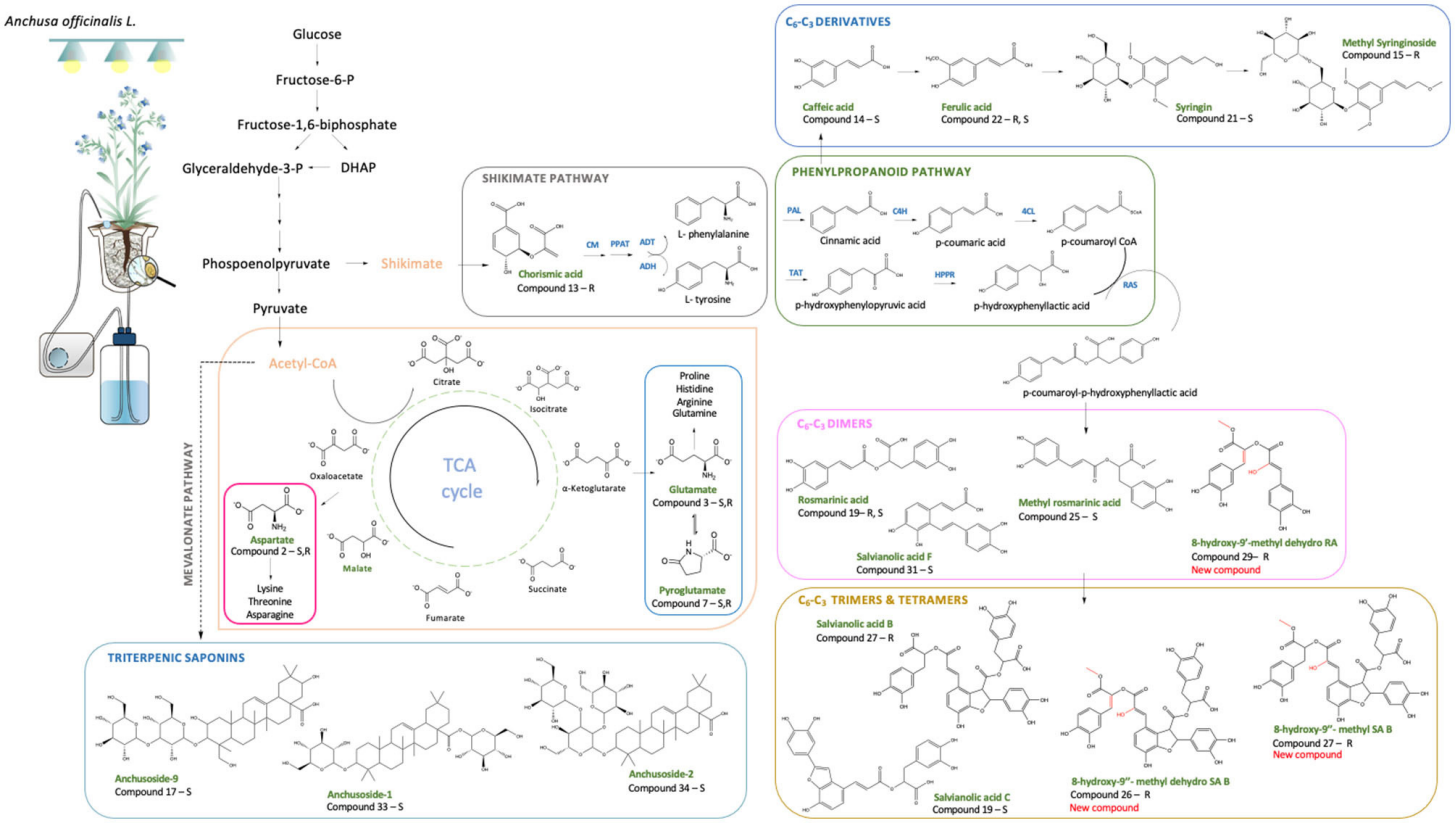
Schematic representation of the main PMs and SMs influenced by the AMF-plant symbiosis in relation to different biosynthetic pathways. Upregulated compounds are reported in green accompanied by the letters R and/or S, indicating whether they were found upregulated in roots and/or shoots, respectively (CM, chorismate mutase; PPAT, prephenate aminotransferase; ADT, arogenate dehydratase; ADH, arogenate dehydrogenase; PAL, phenylalanine ammonia-lyase; TAT, tyrosine aminotransferase; C4H, cinnamic acid 4-hydroxylase; HPPR, hydroxyphenylpyruvate reductase; 4CL, 4-coumaric acid coenzyme A-ligase; RAS, rosmarinic acid synthase; HXL, hydroxylase).

Regarding the primary metabolism, 10 PMs were significantly upregulated in both shoot and root parts of the plants. An increased accumulation of specific amino acids, such as asparagine, glutamic, and pyroglutamic acids, and organic acids, such as DL-malic, was observed in M plants with a *fold* change increment varying from 6.29 to 8.39 in the roots and 2.19 to 9.24 in the shoots. Primary metabolic reprogramming was particularly observed in root tissues at Exp. 1, after 9 days in the S-H cultivation system, while no significant differences were assessed between M and NM plants in Exp. 2 after 30 days. In more details, Volcano-plots evidenced pyroglutamic acid as the most upregulated compound, showing a *fold* change of 8.39 and 9.24 in shoots and roots, respectively. Pyroglutamic acid (PG), also known as 5-oxoproline, is a natural amino acid derivative in which the free amino group of glutamic acid cyclizes to form a lactam (Schliemann et al., 2008; Jiménez-Arias et al., 2019). This PM is an important reservoir and analog of glutamate (Glu) (Kumar and Bachhawat, 2011; Jiménez-Arias et al., 2019), which was also increased in M plants (Table 3a). Glutamate is considered as the precursor amino acid of glutamine, histidine, arginine, and proline (Figure 6). Therefore, the accumulation of glutamate and PG could reflect the intense metabolic activity occurring during the growth and development of M plants. Interestingly, high accumulations of Glu in M plants have been reported as a significant nitrogen pool, reflecting the ability of AMF to enhance N assimilation in plants (Bücking et al., 2012; Zeng et al., 2013; Rivero et al., 2015). N movement in AMF symbiosis includes the uptake of N by the fungal extraradical mycelium, Arg synthesis in the extraradical mycelium, and transport to the intraradical mycelium, where it is broken down to release N for transfer to the host plant (Zhu et al., 2016). Moreover, AMF are capable of accessing organic nitrogen-containing compounds directly from the soils, resulting in an improved plant uptake of multiple amino acids: phenylalanine, lysine, asparagine, arginine, histidine, methionine, tryptophan, and cysteine (Whiteside et al., 2012). Zhu et al. (2016) reported that AMF symbiosis increased the amino acid concentrations and altered the amino acid profile of maize plants under low temperature stress, but, still, the mechanism behind the AMF-mediated changes in amino acid profile is unknown and merits further studies. More recently, Jiménez-Arias et al. (2019) have reported a stress-tolerant effect of PG in crops grown under environmental stress conditions, suggesting that the excessive upregulation of this PM in our S-H cultivation system could also result from an adaptation of the plant-AMF associates to the specific environmental conditions encountered during the short-term experiment. As reported for the changes of the phenotypic traits, the initial stress encountered in Exp. 1 could have played a role in the boosting of metabolism of M plants.

Malic acid levels also significantly increased in the M plants, with a *fold* change increment of 8.17 and 7.79 in shoots and roots, respectively. This organic acid is one of the main intermediates of tricarboxylic acid (TCA) cycle and precursor of aspartic acid (Asp) (Figure 6), which was also increased in the M plants. The latter is an important intermediate precursor of other amino acids, such as asparagine, threonine, lysine, isoleucine, and methionine, and is mainly present in the early development stage of the plant, while its decrease is a consequence of the relative increase of derived amino acids as the plant matures (Wang and Larkins, 2001).

Similar to PG, Glu, and malic acid (MA), all identified amino acids showed a higher accumulation in M plants (Table 3a). These aspects are in accordance with our phenotypic traits observations and indicate that the primary metabolism is strongly affected by the AMF symbiosis (Rivero et al., 2015). However, in Exp. 2, the well-established AMF symbiosis occurred at day 30 of the S-H cultivation system did not affect PMs concentration and neither the TFW of M plants. In all cases, we found that no phenotypic differences between plants lead to a common PM content, reflecting the importance of the growth stage in the production of specific PMs. In our analysis, we hypothesized that the significant effect of the AMF symbiosis, mainly occurring at the early growth stage in the S-H system, was related to the stress conditions of plant establishment in pots and at the transfer to the S-H cultivation system. However, further investigations are needed to fully understand the impact in later growth stages.

Besides the aforementioned amino acids and organic acids widely present in living organisms, a natural auxin, phenylacetic acid (PAA), was found significantly upregulated in the roots of M plants at the end of Exp. 1 (*fold* change of 5.28). With a less restricted meaning of PMs, the natural auxin PAA plays a key role in plant-growth promotion (Hammad et al., 2003). It has been reported as more effective than indole-3-acetic acid (IAA) in the induction of lateral root growth and for carrying cellular growth (Cook, 2019). Auxins are also particularly essential at early growth stages in host-roots (Fusconi, 2014) to ensure successful colonization by AMF since the root architecture is subject to continuous changes by increasing branching and development of lateral roots (Fusconi, 2014; Cook, 2019). This may contribute to PAA accumulation in the roots of M plants in Exp. 1. However, as the plants adapt to the S-H system, no statistical difference in PAA content was observed between M and NM plants at the end of Exp. 2.

##### Impact on Secondary Metabolism

Regarding the secondary metabolism, 24 different SMs were significantly up and downregulated in shoots and roots of AMF-colonized plants, with a *fold* change varying from 1.52 to 6.90. As reported for PMs, SMs were essentially upregulated in the root system of M plants in Exp. 1 after 9 days of growth in the S-H system. In particular, 12 compounds were exclusively accumulated in roots, 9 in shoots, while 2 were identified in both parts of the M plants. Although the increment involved mainly phenolic compounds, colonization by AMF also impacted the expression of glycosylated triterpenoids in M shoots.

In total 15 phenylpropanoids, among which 10 have been tentatively identified as di-, tri-, or tetramers of C_6_-C_3_ compounds (e.g., rosmarinic acid, salvionolic acid C, and salvianolic acid B), were significantly upregulated in M plants. Eight of them were upregulated exclusively in the roots, 5 in the shoots, while 2 in both parts. The structures of the aforementioned compounds were assigned according to their pseudomolecular ions and MS/MS spectra. Interestingly, most phenylpropanoids shared common MS/MS ions at *m*/*z* 179 and at *m*/*z* 135, corresponding to the typical fragments of the caffeoyl moiety (Table 3b). This may also explain their simultaneous upregulation in M plants. Therefore, C_6_-C_3_ compounds and their di-, tri-, and tetrameric derivatives are affected by the AMF symbiosis, mainly in M roots, presenting a *fold* change, ranking from 1.99 to 7.17.

An increased production of chorismic acid was also noticed in the roots of M plants. This compound is considered a precursor of hydroxycinnamic acids (C_6_-C_3_ skeleton) (Figure 6) and thus of the ester derivatives of caffeic acid detected in our study (Table 3b). In support of this increment, previous studies have reported increasing levels of transcripts encoding for the biosynthetic enzyme phenylalanine ammonia lyase (PAL) in mycorrhized plants. This may further confirm our findings since PAL is involved in the formation and accumulation of phenolic acids *via* the phenylpropanoid biosynthetic pathway (Rivero et al., 2015). Indeed, AMF symbiosis elicited signaling cascades, which cause the activation of specific biosynthetic defense pathways, resulting in the release of specific compounds such as polyphenolic derivatives (Bulgakov et al., 2012; Srivastava et al., 2016).

Special attention was given to compound 26 due to its high accumulation in the roots of M plants (*fold* change increment of 7.17). Interestingly, this compound represents a previously undescribed natural product tentatively identified as 8-hydroxy-9”methyl-dehydro-SA B_._ Similarly, compound 27 was assigned as a new C_6_-C_3_ tetramer (*fold* change increment of 3.4) tentatively characterized as 8-hydroxy-9”methyl-SA B. In our analysis, we also identified several dimeric C_6_-C_3_ forms, including the commonly known rosmarinic acid [*m/z* 359.0777 (M-H)^−^, compound 19], widely distributed in *Anchusa* spp. (Kuruuzum-Uz et al., 2012; Dresler et al., 2017; Boskovic et al., 2018) and its new derivative 8-hydroxy-9'-methyl dehydro RA (compound 29). Structures of compounds 26, 27, and 29 are in line with Liu et al. (2007) and with Grzegorczyk-Karolak et al. (2019), showing a common position of unsaturation at carbons C-7” and C-7' for compounds 26 and 29, respectively. Moreover, they exhibit a common methylation at C-9' for compound 29 and at C-9” for compounds 26 and 27, and hydroxylation at C-8 for compounds 29 and at C-8' for compounds 26 and 27.

According to Table 3b, compound 16 represents the only downregulated SM in M plants, showing a *fold* change decrement of 2.28 and 3.07 in both shoot and root tissues, respectively. Based on HRMS data, compound 16 was structurally annotated as lithospermic acid. Interestingly, and as opposed to lithospermic acid, compound 23, identified as the methylated derivative of lithospermic acid at position 9”, showed significant upregulation in the roots of M plants (*fold* change of 2.79). During our analysis, we identified a total of 15 C_6_-C_3_ SMs showing significant upregulation in M plants; among which 7 were tentatively assigned as methyl derivatives. This could suggest that AMF symbiosis induced changes in host plant metabolism (Lohse et al., 2005). In line with our results, previous studies of *R. irregularis* genome has underlined the expression of different methyltransferases (Sun et al., 2019) involved in the methylation of specific compounds, such as phospholipids (Wewer et al., 2014; Feng et al., 2020) in AMF-colonized roots. However, to the best knowledge of the authors, this is the first time that the impact of AMF on SM methylation was reported.

In addition*, p*-Hydroxybenzoic acid (HBA) and syringic acid (SA) were highly accumulated in M roots (*fold* change increment of 6.90 and 4.55, respectively) (Table 3b). HBA and SA are aromatic compounds with a C_6_-C_1_ skeleton synthesized *via* the shikimate pathway involving different C_6_-C_3_ intermediates, the main category of accumulated molecules found in our study (Figure 6) (Widhalm and Dudareva, 2015; Srinivasulu et al., 2018). Both HBA and SA are crucial precursors of a wide variety of essential molecules (e.g., folic acids and ubiquinone) playing a key role in plant fitness (Widhalm and Dudareva, 2015). Their production is often related as a reaction to biotic/abiotic stresses (Widhalm and Dudareva, 2015; Zubek et al., 2015). The accumulation of these compounds in the roots of M plants grown in our S-H cultivation system is in line with the studies of Kara et al. (2015) and Zubek et al. (2015), showing higher abundance of benzoic acids and, especially, of HBA in AMF-colonized plants.

Besides the increment in phenolic compounds, AMF symbiosis also impacted the expression of glycosylated triterpenoids, such as the oleanane-type saponins. Saponins are essentially found in cell membranes of dicotyledonous plants, and they are mainly involved in plant protection mechanisms (Xie et al., 2018). Wide diversity of chemical structures is reported in the plant kingdom on the basis of aglycone structure and the nature of glycosylation (Mugford and Osbourn, 2013). Their content is influenced by various biotic stimuli, such as herbivory and pest attack or related to plant-microorganism symbiosis (Hussain et al., 2017). Interestingly, the ability to produce these compounds has been already reported for *Anchusa* spp., specifically oleanane-type glycosidic derivatives mainly present in the foliar part of the plants (Koz et al., 2009; Chen et al., 2017). In our analysis, and in opposition to phenolic compounds, saponins 17, 32, 33, and 34 showed maximum accumulation in M shoots, with a *fold* change increment ranking from 1.73 to 2.26. Their structures were assigned on the basis of their pseudomolecular ions under a negative ionization mode and by their specific MS/MS fragmentation pattern. Anchusoside-2, as well as Anchusoside-1 and Anchusoside-9, is widely reported for *A. officinalis*. However, this is the first report showing that oleanane-type saponins can be upregulated in shoots of M plants.

Despite the pronounced metabolic variations of Exp. 1, no significant differences were observed at the end of Exp. 2. For instance, after 30 days, neither the SMs shoots and roots content of M plants nor their phenotypic traits showed any significant variation when compared with the NM plants. It is worth mentioning that AMF symbiosis remains active at the end of Exp. 2 through the presence of numerous arbuscules. Nevertheless, no benefits from the symbiosis were noticed for M plants from the phenotypic and the metabolic point of view. Taking into consideration this observation, we can suggest that the AMF impact is considered particularly important in the early growth stage of the plants in the pots and S-H system and, apparently, less effective as it grows older. However, the effect of the growth environment offered by the S-H cultivation system should be carefully considered. Indeed, the limited space could exert an inhibitory effect on growth, which gradually increased in relation to the plant development. Finally, the decrease in the content of the minerals (Exp. 2, T4) could have diminished/limited the AMF-plants nutrient requirements. AMF might have tried to allocate energy mostly on the arbuscules formation in order to optimize the uptake of the remaining nutrients (Le Pioufle et al., 2019).

Interestingly, several of the upregulated secondary metabolites found during our analysis, as a result of the *A. officinalis-*AMF symbiosis, have shown important biological proprieties. For instance, phenylpropanoids and their derivatives, which represent the class of secondary metabolites more affected by the AMF symbiosis, have gained attention due to their low toxicity and a wide array of beneficial effects on human health and disease management. For instance, salvianolic acids showed an important impact on cancer treatment and alleviation of fibrosis disease as well as a good therapeutic effect on cardiovascular and neural protection (Ma et al., 2019). Furthermore, other major affected compounds, such as rosmarinic acid, ferulic acid, caffeic acids, and derivatives, present several health-related properties, such as antioxidant, anti-inflammatory, and antimicrobial activities (Boskovic et al., 2018; Luo et al., 2020). In addition to phenolic compounds, saponins present antibacterial, antifungal, and antiviral properties and are extensively used beyond pharmaceuticals for their surfactant properties (Mugford and Osbourn, 2013). Therefore, AMF symbiosis increases *A. officinalis* performance in the production of several bioactive compounds and lays the foundation for further exploitation of these root symbionts in the manipulation of medicinal plants.

#### Plant Exudates

Despite the fact that AMF symbiosis has been proven to affect different metabolic and physiological processes in plants, the literature related to the impact of AMF on plant exudation is still scarce. It is well-known that plants exude large diversity of compounds, especially SMs, that contribute to the plant fitness by interacting with the surrounding soil microbiota or by playing a role in nutrient acquisition by boosting soil nutrient bioavailability (Voges et al., 2019). In the present study, we took advantage of the controlled environment offered by the S-H cultivation system to investigate any possible differences among plant exudates of M and NM plants. During our analysis, the same Hoagland solution ^dil100X^ percolated through M and NM plants for several weeks, without renewing, in order to compare the plant exudation at 9 and 30 days of the experiment.

Unlike our previously reported findings showing a pronounced metabolic variation between M and NM plants at T1 (Exp. 1), data resulting from the PCA analysis of plant exudates at the same time period showed no differences between M and NM plants. Conversely, upregulation of two mass signals attributed to the coumarin scoparone (compound 35) and the furanocoumarin byakangelicin (compound 36) were identified in the exudates of M plants at T4 of Exp. 2 with a *fold* change increment of 3.21 and 9.50.

Coumarins are plant-derived natural products synthesized *via* the phenylpropanoid pathway and are widely reported as exuded substances involved in defense mechanisms against pathogens (Harbort et al., 2020). Recently, however, these compounds have been also described for their ability to mobilize iron from deprived soil. For instance, their exudation from roots was induced under Fe limitations, aiming for more efficient iron acquisition (Chutia et al., 2019). In addition, similar studies reported that AMF symbiosis could induce the accumulation of coumarins in the radical part of mycorrhized plants for their secretion in the rhizosphere under Pi limitation (Stringlis et al., 2019). This hypothesis could explain our findings on plant exudates since at T4 clear depletion of Pi and ${\text{NO}}_{3}^{-}$was observed.

## Conclusion

In conclusion, the AMF colonization influenced specific plant's biosynthetic pathways resulting in a qualitative and quantitative modification of different metabolites production. Colonization by the AMF was followed by an enhanced production of PMs, including organic acids (involved in the energy pathways of the eukaryotic cell) and key amino acids, with the potential to act as precursors of other amino acids and as building blocks for the production of macromolecules. Furthermore, SMs production was significantly affected, especially the phenolic compounds and the oleanane-types glycosidic derived from the phenylpropanoid and mevalonate pathways, respectively. In total, 16 C_6_-C_3_ caffeic acid derivatives were induced mainly in the roots of M plants while 4 oleanane-types saponins were accumulated in the shoot parts. Besides the well-documented C_6_-C_3_ phenolics herein we noticed, for the first time, the production of two new derivatives of SA and one new derivative of RA, all presenting a common substitution pattern (methylation and hydroxylation) in the roots of M plants. Interestingly, upregulation of methylated compounds was underlined in AMF-colonized plants, suggesting that these fungi have the potential to alter the plant biosynthetic pathways and to induce the production of different compounds. It will be of great interest to understand the molecular mechanisms behind the accumulation of specific compounds in the presence of AMF and how this could potentially be translated in increasing the production of specific compounds, which were valuable for human purposes.

## Data Availability Statement

The datasets presented in this study can be found in online repositories. The names of the repository/repositories and accession number(s) can be found in the article/Supplementary Material.

## Author Contributions

AC: experimental setup, data collection, analysis and interpretation, drafting the work, commentaries, corrections, final approval, and agreement with all aspects of the work. ET: data collection, analysis and interpretation, drafting the work, commentaries, corrections, final approval, and agreement with all aspects of the work. NT: contribution to the experimental setup and data collection, data analysis and interpretation, draft corrections, final approval, and agreement with all aspects of the work. IL: contribution to the development of the experiment, draft correction, and final approval and agreement with all aspects of the work. AT: data analysis and interpretation, final approval, and agreement with all aspects of the work. MM: contribution to the experimental setup, data collection, final approval, and agreement with all aspects of the work. NF: draft correction, final approval, and agreement with all aspects of the work. SD: substantial contributions to the conception and design of the experiments, interpretation of the data, draft corrections, final approval, and agreement with all aspects of the work. All authors contributed to the article and approved the submitted version.

## Conflict of Interest

The authors declare that the research was conducted in the absence of any commercial or financial relationships that could be construed as a potential conflict of interest.

## Publisher's Note

All claims expressed in this article are solely those of the authors and do not necessarily represent those of their affiliated organizations, or those of the publisher, the editors and the reviewers. Any product that may be evaluated in this article, or claim that may be made by its manufacturer, is not guaranteed or endorsed by the publisher.

## Footnotes

^1^https://www.mycorrhiza.be/ginco-bel/.
