# Supplementary material for: The Arbuscular Mycorrhizal Fungus Rhizophagus irregularis MUCL 41833 Modulates Metabolites Production of Anchusa officinalis L. Under Semi-Hydroponic Cultivation
Source: Front Plant Sci. 2021 Sep 1;12:724352. doi: 10.3389/fpls.2021.724352 (PMC8443025; doi:10.3389/fpls.2021.724352)
Supplement: Supplementary file 1 [file Data_Sheet_1.docx]

**Supplementary materials**

**Supplementary Figure 1.** Partial least square analysis – Discriminant analysis (PLS-DA) and permutation test (100 rearrangements) – Comparison of UHPLC-HRMS metabolic profiles from M and NM root (**A_1_** and **A_2_**) and shoot (**B_1_** and **B_2_**) samples after 1 week (T1) of growth in the S-H cultivation system (M_T1: green dots; NM_T1: red dots).


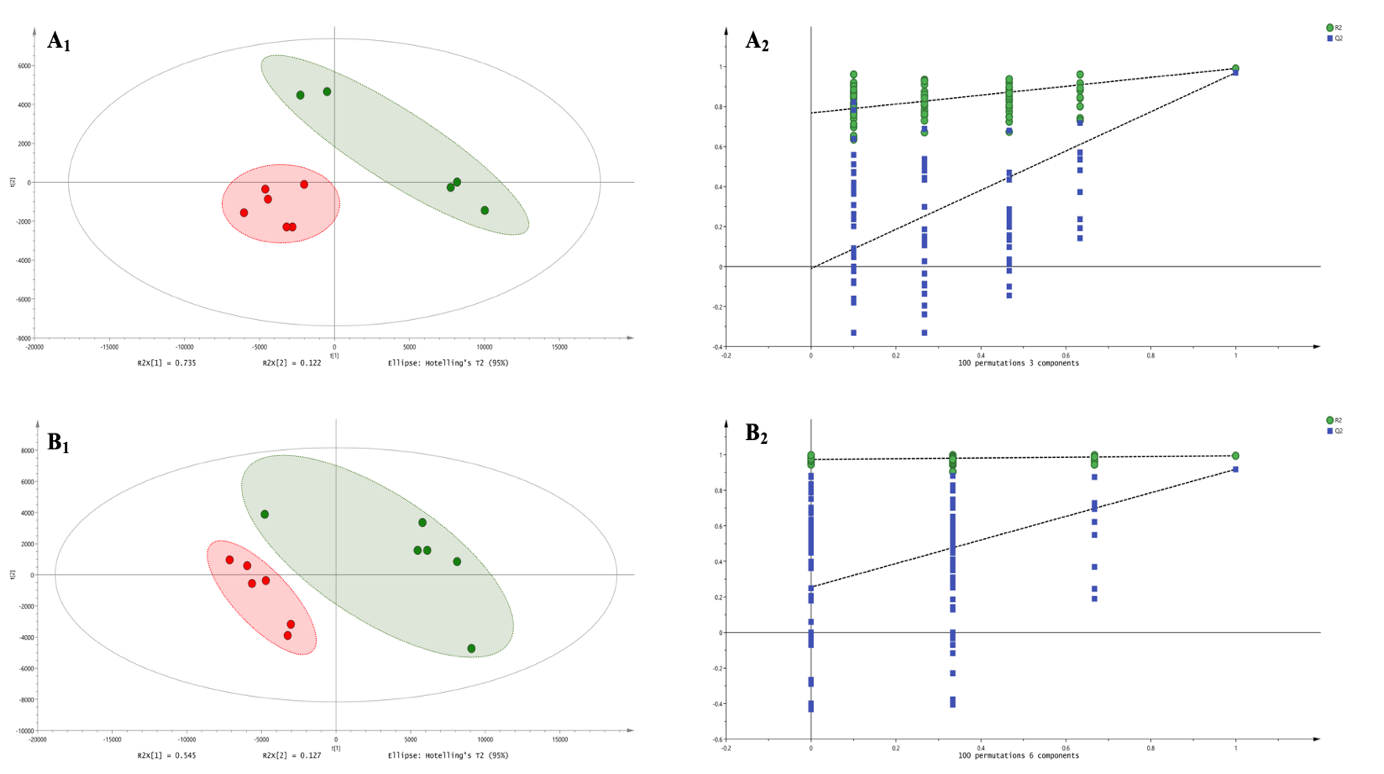


**Supplementary Figure 2.** Boxplots illustrate the means of Pi **(A)** and NO_3_^-^ **(B)** concentration in the nutrient solutions of M and NM plants at four different time sampling (day 9 – T1; day 15 – T2; day 22 – T3 and day 30 -T4). The presence of * indicates a significant difference at T4, according to the pairwise comparison test (p < 0.05). Means of the single time are averaged between the two treatments (M and NM).


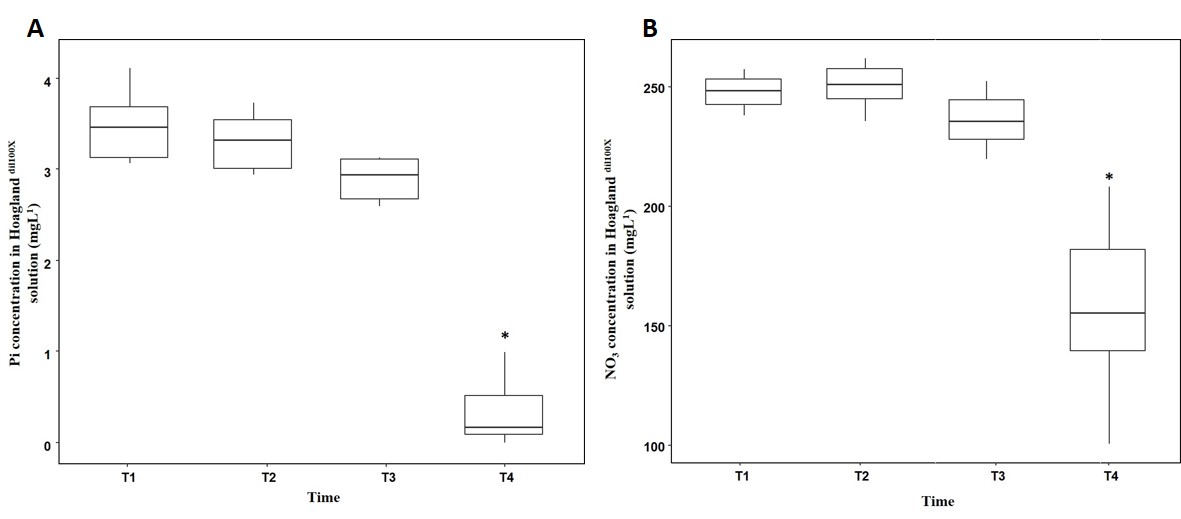


**Supplementary Figure 3.** Partial least square analysis – Discriminant analysis (PLS-DA) and permutation test (100 rearrangements) – Comparison of UHPLC-HRMS metabolic profiles from M and NM root (**A_1_** and **A_2_**) and shoot (**B_1_** and **B_2_**) samples after 4 weeks (T4) of growth in the S-H cultivation system (M_T1: green dots; NM_T1: red dots).


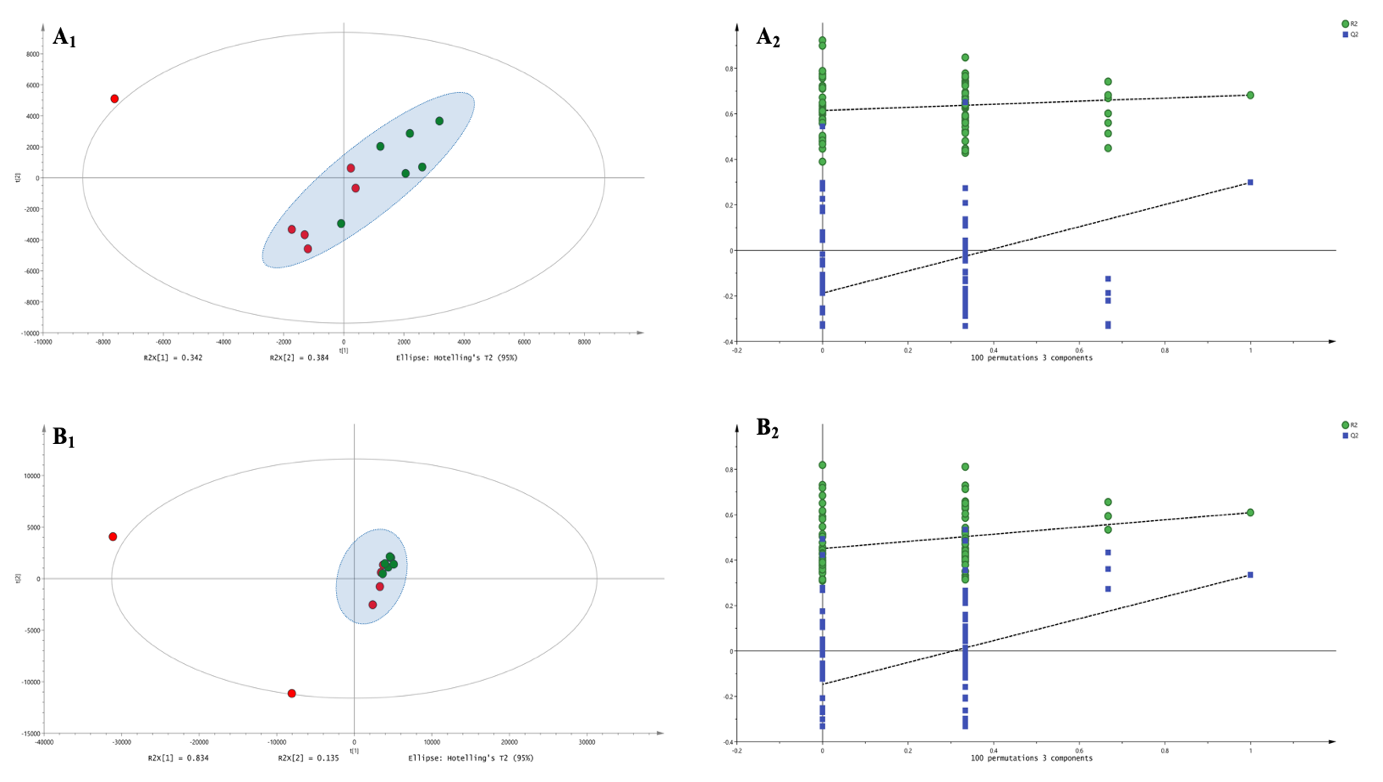


**Supplementary Figure 4.** Partial least square analysis – Discriminant analysis (PLS-DA) and permutation test (100 rearrangements) – Comparison of UHPLC-HRMS metabolic profiles from M and NM nutrient solution samples after 1 (Exp. 1 - T1) and 4 weeks (Exp. 2 - T4) of growth in the S-H cultivation system (M_T1: blue dots; NM_T1: yellow dots; M_T4: green dots; NM_T4: red dots).


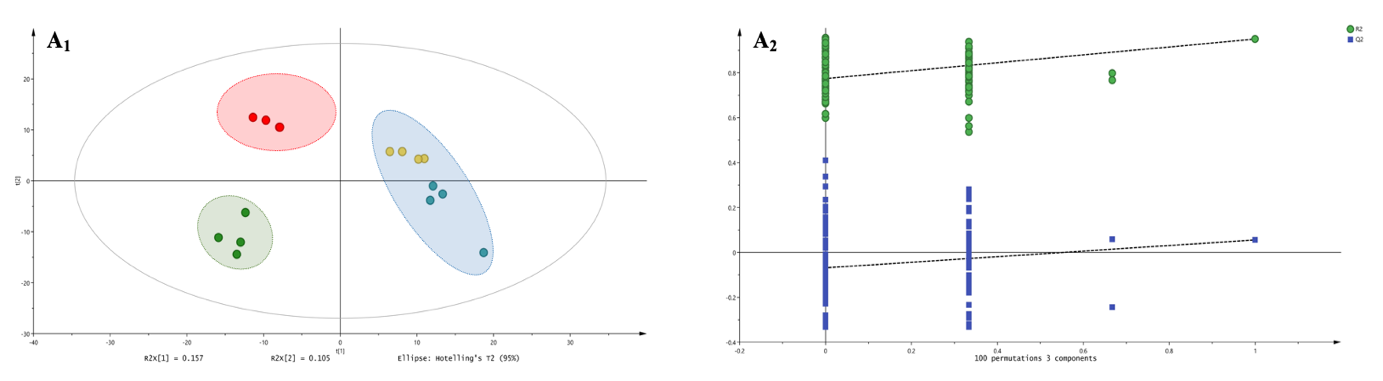


**Supplementary Figure 5.** Volcano-plot analysis: Up and downregulation of PMs and SMs between M and NM nutrient solutions after 4 weeks (T4) of growth in the S-H system. The statistically significant compounds (p < 0.05 & FC > 1.5), upregulated in M nutrient solutions, were represented in green in the upper right-side part of the plot.


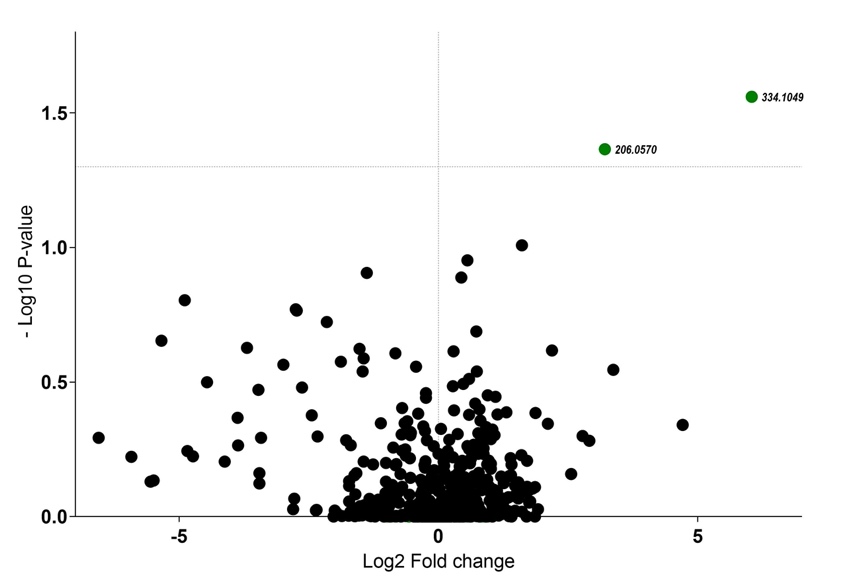


**Supplementary Figure 6.** Volcano-plot analysis – Up and downregulation of statistically significant compounds (p < 0.05 & FC > 1.5) between nutrient solutions of M plants after 1 (Exp. 1 - T1) and 4 weeks (Exp. 2 - T4) of S-H cultivation system. Upregulated compounds at T4 of experiment were represented in green in the right side of the plot, while upregulated compounds at T1 of experiment were represented in red in the left side of the plot.
